# Differential STAT gene expressions of *Penaeus monodon* and *Macrobrachium rosenbergii* in response to white spot syndrome virus (WSSV) and bacterial infections: Additional insight into genetic variations and transcriptomic highlights

**DOI:** 10.1371/journal.pone.0258655

**Published:** 2021-10-15

**Authors:** Tze Chiew Christie Soo, Subha Bhassu

**Affiliations:** 1 Faculty of Science, Animal Genetics and Genome Evolutionary Laboratory (AGAGEL), Department of Genetics and Molecular Biology, Institute of Biological Sciences, University of Malaya, Kuala Lumpur, Malaysia; 2 Terra Aqua Laboratory, Centre for Research in Biotechnology for Agriculture (CEBAR), Research Management and Innovation Complex, University of Malaya, Kuala Lumpur, Malaysia; UCSI University, MALAYSIA

## Abstract

Diseases have remained the major issue for shrimp aquaculture industry for decades by which different shrimp species demonstrated alternative disease resistance or tolerance. However, there had been insufficient studies on the underlying host mechanisms of such phenomenon. Hence, in this study, the main objective involves gaining a deeper understanding into the functional importance of shrimp STAT gene from the aspects of expression, sequence, structure, and associated genes. STAT gene was selected primarily because of its vital signalling roles in stress, endocrine, and immune response. The differential gene expressions of *Macrobrachium rosenbergii* STAT (*MrST*) and *Penaeus monodon* STAT (*PmST*) under White Spot Syndrome Virus (WSSV) and *Vibrio parahaemolyticus*/*Vp*_AHPND_ infections were identified through qPCR analysis. Notably, during both pathogenic infections, *MrST* demonstrated significant gene expression down-regulations (during either early or later post-infection time points) whereas *PmST* showed only significant gene expression up-regulations. Important sequence conservation or divergence was highlighted through STAT sequence comparison especially amino acid alterations at 614 aa [K (Lysine) to E (Glutamic Acid)] and 629 aa [F (Phenylalanine) to V (Valine)] from PmST (AY327491.1) to PmST (disease tolerant strain). There were significant differences observed between in silico characterized structures of MrST and PmST proteins. Important functional differentially expressed genes (DEGs) in the aspects of stress, endocrine, immune, signalling, and structural were uncovered through comparative transcriptomic analysis. The DEGs associated with STAT functioning were identified including inositol 1,4,5-trisphosphate receptor, hsp90, caspase, ATP binding cassette transmembrane transporter, C-type Lectin, HMGB, ALF1, ALF3, superoxide dismutase, glutathione peroxidase, catalase, and TBK1. The main findings of this study are STAT differential gene expression patterns, sequence divergence, structural differences, and associated functional DEGs. These findings can be further utilized for shrimp health or host response diagnostic studies. STAT gene can also be proposed as a suitable candidate for future studies of shrimp innate immune enhancement.

## 1.0 Introduction

The shrimp aquaculture industry is an important global economic sector especially for some middle- or low-level economies [1]. Vital shrimp species including *Litopenaeus vannamei*, *Penaeus monodon*, and *Macrobrachium rosenbergii* had majorly contributed to the annual global shrimp aquaculture production of roughly 6.0 million tonnes in year 2018 [2]. However, the shrimp aquaculture production had always been significantly impeded by various shrimp diseases which remained the top concern for aquaculture farmers [3]. These shrimp diseases are mainly caused by viruses and bacteria by which the most serious diseases are White Spot Disease (WSD), Acute Hepatopancreatic Necrosis Disease (AHPND), Hepatopancreatic Microsporidiosis, Yellow Head Disease, and Infectious Myonecrosis [4].

WSD is a highly lethal shrimp viral disease caused by White Spot Syndrome Virus (WSSV). WSSV is an enveloped, rod-shaped, tailed, and double-stranded DNA virus [5] classified under family Nimaviridae and genus Whispovirus [6]. WSSV had emerged in China since year 1992 and spread to other Asia and America regions [7,8]. This disease can lead to 100% mortality within 3–10 days post-infection [5]. The host range of WSSV includes *P*. *monodon*, *L*. *vannamei*, *M*. *rosenbergii*, and other Penaeid shrimp species. Some clinical signs of WSD are lethargy, white spot formation on muscle, and reduced appetite [5].

In addition, AHPND is a serious shrimp bacterial disease caused by a pathogenic strain of Gram-negative *Vibrio parahaemolyticus* bacteria known as *Vp*_AHPND_ [9,10]. The *Vp*_AHPND_ bacteria contain a 70-kbp plasmid (pVA1) capable of producing *Photorhabdus* insect-related (Pir) toxins, PirA and PirB [9]. AHPND had emerged in China since year 2009 which then spread to Southeast Asia and Mexico regions [11,12]. This disease can cause 40–100% mortality within early 35 days shrimp post-stocking [13]. AHPND disease outbreaks had been reported for *P*. *monodon*, *L*. *vannamei*, and *Fenneropenaeus chinensis* [14]. The gross clinical signs of AHPND disease include slow growth, lethargy, pale and atrophied hepatopancreas, empty stomach, and empty gut [13]. Intriguingly, despite being susceptible to Vibriosis disease caused by *V*. *parahaemolyticus* bacteria [15], *M*. *rosenbergii* under salinity condition of 20 ppt or lower was shown to be not susceptible to AHPND infection [16].

Different strategies are applied in shrimp disease prevention which include selective breeding [17], antibiotics usage [18], probiotics usage [19], and proper biosecurity measures [17]. For selective breeding, it is important to ensure the successful accumulation of desired genetic variants associated with stronger immune response. Among the crucial shrimp innate immune genes, Janus Kinase (JAK)-Signal Transducer and Activator of Transcription (STAT) pathway components can be specially highlighted due to its important and diverse functioning, for example, in immunity [20,21], neuron signalling [22], endocrine [23], stress-induced cell survival [24], and metabolism [25]. JAK-STAT pathway activation involves initial cytokine recognition by the receptor, followed by receptor dimerization, JAK phosphorylation, STAT phosphorylation and dimerization, and lastly translocation of dimerized STAT to the nucleus for gene expression regulation [21,26]. Seven mammalian STATs had been determined, including STAT1, STAT2, STAT3, STAT4, STAT5A, STAT5B, and STAT6 [21]. Although less studied compared to model organisms, shrimp JAK-STAT pathway components such as JAK, STAT, Suppressors of Cytokine Signalling (SOCS), and Protein Inhibitor of Activated STAT have also been identified through various research efforts in the past years [27–29].

Furthermore, there had been some studies on the STAT gene expression changes in different shrimp species after pathogenic infections. STAT gene expression was up-regulated in *Fenneropenaeus chinensis* challenged with WSSV and *Vibrio anguillarum* [30]. The significant up-regulation of *Marsupenaeus japonicus* [31] and *L*. *vannamei* [32] STAT gene expressions were identified under WSSV infection. Nevertheless, under some diseased conditions, *Macrobrachium* spp. also demonstrated non-differential STAT gene expression. For example, *M*. *nipponense* had no significant STAT gene expression changes after *Aeromonas hydrophila* [33] and non-O1 *Vibrio cholerae* [34] bacterial infections. Interestingly, due to the immune signalling importance of STAT gene, there exists risk of shrimp STAT manipulation by the invading pathogens as demonstrated by the shrimp STAT hijacking by WSSV virus [35].

The study of gene expression is an efficient strategy for the fast and accurate determination of gene activation or repression during pathogenic infections. Real time quantitative PCR (qPCR) and RNA-Seq analyses are more commonly utilized for gene expression studies in recent decades [36,37]. qPCR involves the usage of intercalating dyes or probes and qPCR machine for the determination of gene expression fold change between different treatment groups [38]. RNA-Seq utilizes high throughput next-generation sequencing (NGS) technology and has advantages in terms of price, efficiency, difficulty, and application range compared to more traditional methods such as microarray [39].

Despite the increasing numbers of gene expression studies involving pathogen-challenged shrimps, there had been a lack of research focusing on the comparison of immune gene expressions across different pathogenic conditions especially STAT gene. Therefore, this study involved identification and comparison of differential gene expressions of *M*. *rosenbergii* STAT (*MrST*) and *P*. *monodon* STAT (*PmST*) upon WSSV and *V*. *parahaemolyticus*/*Vp*_AHPND_ infections. STAT gene was selected mainly because of its diverse functional importance through JAK-STAT pathway. This was followed by sequence and structure divergence identification between MrST and PmST. This is because genetic variations can lead to significant gene expression changes and functional alterations during pathogenic infections. A comparative transcriptomic analysis was also conducted to elucidate the underlying stress, endocrine, immune, signalling, and structural DEGs associated with STAT gene functioning during pathogenic infections. Overall, this study had the aim of obtaining more information on the functional importance of shrimp STAT genes involved in the aspects of expression, sequence, structure, and associated genes. The aim was successfully achieved.

## 2.0 Materials and methods

### 2.1 Pathogen preparations

For WSSV virus propagation, the feeding of local *P*. *monodon* shrimps (15–20 g body weight) with WSSV-infected shrimp muscle tissues was conducted. The moribund shrimps were confirmed to be WSSV positive through PCR [40] and stored at -80°C. WSSV virus stock solution was then prepared [41] which involved the homogenization and lysis of the WSSV-infected shrimp muscle tissues in TN Buffer followed by centrifugation, filtration, and storage at -80°C. The WSSV stock solution viral copy number was quantified using primer pairs VP28-140Fw and VP28-140Rv [42].

On the other hand, *P*. *monodon* suspected with AHPND outbreak were collected and validated through both clinical sign observation and AP3 PCR detection method [43]. The *Vp*_AHPND_ bacteria [44] were selectively propagated by incubating the digestive organs of *Vp*_AHPND_-infected shrimps in the order of tryptic soy broth (TSB+), thiosulfate citrate bile salt (TCBS) agar, and tryptic soy agar (TSA+). The bacteria preservation was done through cryovials (CRYOBANK^™^) at -80°C and utilized for downstream experiments.

### 2.2 Pre-challenge works

For the WSSV and *V*. *parahaemolyticus* challenge with *Macrobrachium rosenbergii*, *M*. *rosenbergii* juvenile prawns (5–8 g body weight) were purchased from a hatchery at Kuala Kangsar, Perak, Malaysia. The acclimatization of the prawns was conducted for seven days under aseptic experimental setup. Each tank contained 10 prawns with aerated freshwater at 28 ± 1.0°C.

Whereas for WSSV challenge with *P*. *monodon*, locally obtained juvenile 4^th^ generation *P*. *monodon* shrimps (15–20 g body weight) of Mozambique, Africa strain (10 shrimps per tank) were acclimatized for seven days under aseptic experimental setup with aerated artificial seawater (30 ppt) at 28 ± 1.0°C. For the *Vp*_AHPND_ experimental challenge, disease tolerant crossbred (13^th^ generation Madagascar strain with 5^th^ generation local strain) juvenile *P*. *monodon* shrimps (15–20 cm body length) were involved. The acclimatization of the shrimps (27 shrimps each tank) was done for seven days under aseptic experimental setup with aerated artificial seawater (30 ppt) at 28 ± 1.0°C.

The negative screening of the prepared *M*. *rosenbergii* and *P*. *monodon* shrimps was conducted before experimental challenge using PCR methods and confirmed to be WSSV-free [40] and *V*. *parahaemolyticus*/*Vp*_AHPND_-free [43] respectively.

### 2.3 Experimental challenge details

The intramuscular injection of *M*. *rosenbergii* prawns was conducted which involved 100 μl filtered WSSV virus stock solution (1 x 10^5^ copies/ml) (WSSV treatment groups), 100 μl cultured *V*. *parahaemolyticus* (PCV08-7) (1 x 10^5^ cfu/ml) (*V*. *parahaemolyticus* treatment groups), and 100 μl 2% NaCl (w/v) (negative control groups) respectively. The shrimp hepatopancreas was collected at 0, 3, 6, 12, 24, and 48 hours post-infection (hpi) and stored at -80°C. The challenge details were described in previous publications [45,46].

Besides that, for the WSSV experimental challenge, *P*. *monodon* shrimps were injected with 100 μl filtered WSSV stock solution (4.11 x 10^5^ copies/μl). Sterile PBS was injected for the negative control group shrimps. The shrimp hepatopancreas collection was done at 0, 3, 6, 12, 24, and 48 hpi and also 12 days post-infection (dpi) (survivors) and stored at -80°C. The challenge details were described in previous publication [47].

For the *Vp*_AHPND_ experimental challenge, *P*. *monodon* shrimps were infected with *Vp*_AHPND_ bacteria (KS17.S5-1 positive strain) (2 × 10^6^ cfu/ml) [48] based on a modified immersion method [10]. Sterile TSB+ broth was used instead of *Vp*_AHPND_ for the negative control group shrimps. The shrimp hepatopancreas was collected at 0, 3, 6, 12, 24, and 48 hpi and stored at -80°C. The challenge details were also described in previous publication [44].

All tanks were equipped with aerators and water filters. The experimental challenges were conducted with three biological replicates for each treatment and control groups. The positive screening of the challenged shrimps was done through PCR methods for WSSV [40] and *V*. *parahaemolyticus*/*Vp*_AHPND_ [43] confirmation respectively. The University of Malaya granted Ethical approval for the study within its facilities (Ethical Application Ref: S/31012019/26112018-05/R).

### 2.4 Total RNA extraction and first strand cDNA synthesis

Total RNA samples were extracted from shrimp hepatopancreas at each post-infection time interval of both treatment and control groups using NucleoSpin RNA II Extraction Kit (Macherey’s-Nagel, Germany), RNA Isolation Kit (Macherey’s-Nagel, Germany), and TransZol Up Plus RNA Kit (TransGen Biotech, Beijing, China) respectively. The extracted RNA samples were also treated with TransScript^®^ One-Step gDNA Removal and cDNA Synthesis SuperMix (TransGen Biotech, Beijing, China) to achieve DNA contaminant removal and first strand cDNA synthesis for subsequent downstream applications. The manufacturer’s protocols were followed for all kits utilized.

### 2.5 Expression profile comparison through qPCR analysis

The STAT gene expression profiles of *M*. *rosenbergii* (*MrST*) and *P*. *monodon* (*PmST*) during WSSV and *V*. *parahaemolyticus*/*Vp*_AHPND_ infections were determined and compared through quantitative real-time PCR (qPCR) analysis. Three biological replicates with three technical replicates each were applied for every treatment group. The qPCR primers were designed through PrimerQuest Tool software (https://sg.idtdna.com/Primerquest/home/Index) and listed in S1 Table.

The *MrST* qPCR experiments were conducted using TaqMan^®^ Universal PCR Master Mix kit and Step One Plus Real-Time PCR System^®^ instrument (Applied Biosystems, Foster City, CA, USA). The qPCR reaction (20 μl) consisted of 10 μl TaqMan Universal RT-PCR Master Mix, 1 μl primers/probe set containing 900 nM of forward reverse primers, 300 nM probe, 2 μl template cDNA, and nuclease-free water. The qPCR cycling program involved 50°C for 2 mins, 40 cycles of 95°C for 10 mins, 95°C for 15 secs, and 60°C for 1 min. Elongation factor 1-alpha (EF1a) gene was chosen as the internal control reference gene [49]. The experimental protocol details were mentioned previously [50].

The *PmST* qPCR experiments were carried out using GoTaq^®^ qPCR Master Mix kit (Promega, Madison, Wisconsin, USA) and Agilent Technologies Stratagene Mx3005P instrument. The qPCR reaction (20 μl) included 10 μl GoTaq^®^ qPCR 2X Mix, 500 nM forward primer, 500 nM reverse primer, 2 μl template cDNA, and nuclease-free water. The qPCR cycling program of 95°C for 2 mins, 40 cycles of 95°C for 15 s, and 56°C for 35 secs was utilized. EF1a gene was selected as the internal control reference gene as well.

The analysis of the Ct values obtained was conducted through Livak’s 2^ddCt relative quantification method [51]. The differential gene expression values determined were then statistically validated through One-Way ANOVA analysis with post hoc Duncan test using SPSS software Version 22 (Significance value: P<0.05). The post hoc Duncan test was carried out to identify the exact differences of different treatment groups (classified under alphabetical subsets) when One-Way ANOVA analysis was significant. The raw data for the qPCR experiments was shown in S1 Data.

### 2.6 Sequence and structural comparison of STAT genes

The *MrST*, *PmST*, and *LvST* sequences were retrieved from NCBI nucleotide database (NCBI Accession Numbers: **KT380661.1; AY327491.1; HQ228176.1**) [46]. The *MrST* sequence validation was done through PCR method and subsequent Sanger Sequencing analysis. In addition, the *PmST* sequence of disease tolerant *P*. *monodon* shrimps used in this study was determined through PCR technique using conserved site targeting strategy. The PCR primers involved were designed using PrimerQuest Tool software (https://sg.idtdna.com/Primerquest/home/Index) and listed in S2 Table. The PCR experiments were conducted using GoTaq^®^ Flexi DNA Polymerase kit (Promega, Madison, Wisconsin, USA) and Eppendorf Mastercycler EP Gradient S instrument. Each PCR reaction contained 5 μl 5 x GoTaq^®^ Flexi Buffer, 1.5 ul 25 mM MgCl_2_ solution, 0.5 μl 10 mM dNTPs, 0.25 ul GoTaq^®^ DNA Polymerase (5U/μl), 400 nM forward primer, 400 nM reverse primer, 1.2 μl template cDNA, and nuclease-free water. The PCR cycling program involved initial denaturation of 95°C for 5 mins, 40 cycles of 95°C for 45 s, 56.7°C for 45 s and 72°C for 45 s, and final extension of 72°C for 5 mins.

The amplified PCR products were then sent for Sanger Sequencing. The sequencing results were trimmed using MEGA7 [52] and Chromas (https://technelysium.com.au/wp/chromas/) software. The sequence identities and their homologs were confirmed through NCBI homology BLAST search [53]. The translated amino acid sequences of MrST and PmST were obtained using NCBI Open Reading Frame (ORF) Finder (https://www.ncbi.nlm.nih.gov/orffinder/), ExPASy Translate (https://web.expasy.org/translate/), and Show Translation (https://www.bioinformatics.org/sms/show_trans.html). The MrST, PmST, PmST (disease tolerant strain), and LvST nucleotide and translated amino acid sequences were compared using Clustal Omega software. The MrST, PmST, LvST, and other STAT homologous sequences (retrieved from NCBI database) were utilized for phylogenetic analysis at nucleotide and amino acid levels (Maximum likelihood method) through MEGA7 software [52]. For structural comparison, the MrST and PmST (disease tolerant strain) translated amino acid sequences were characterized and compared *in silico* using ProtParam (https://web.expasy.org/protparam/), NCBI Conserved Domain Search (CDD) [54], Protter [55], MultiLoc 2 Predictor [56], RNAFold Web Server [57], PSIPRED [58], PROSITE [59], and SWISS-MODEL [60].

### 2.7 Identification and comparison of annotated differentially expressed genes (DEGs)

For additional identification and validation of STAT-related functional genes, the determination and comparison of Differentially Expressed Genes (DEGs) from *M*. *rosenbergii* and *P*. *monodon* under WSSV and *V*. *parahaemolyticus*/*Vp*_AHPND_ infection conditions were conducted. The DEGs involved were identified and retrieved from RNA-Seq results of associated previous publications [44–47]. The data is available at the NCBI SRA database: **SRR1424572**, **SRR1424574**, **SRR1424575**, and **SRP153251**.

Generally, the extracted *M*. *rosenbergii* and *P*. *monodon* hepatopancreas RNA samples were treated with DNase and subsequently sent for cDNA library preparation and NGS sequencing using Illumina HiSeq 2000/BGI-SEQ 500 Sequencer platform by the Beijing Genome Institute (Hong Kong). The raw sequencing reads were filtered by which the clean reads were used for DEGs determination. This was followed by the functional annotation of the identified DEGs through mapping to different databases. The details of RNA-Seq data analysis were described in previous publications [44–47].

The stress, immune, and endocrine DEGs were mainly identified and compared between different treated samples. The patterns of interaction including co-activation or co-repression of DEGs under different pathogenic conditions especially those involved in STAT functioning were elucidated. The qPCR validation details of these RNA-Seq results were also described in previous publications [44–47].

## 3.0 Results

### 3.1 Differential expression profiles between *M*. *rosenbergii* STAT (*MrST*) and *P*. *monodon* STAT (*PmST*) during pathogenic infections

**Fig 1:**
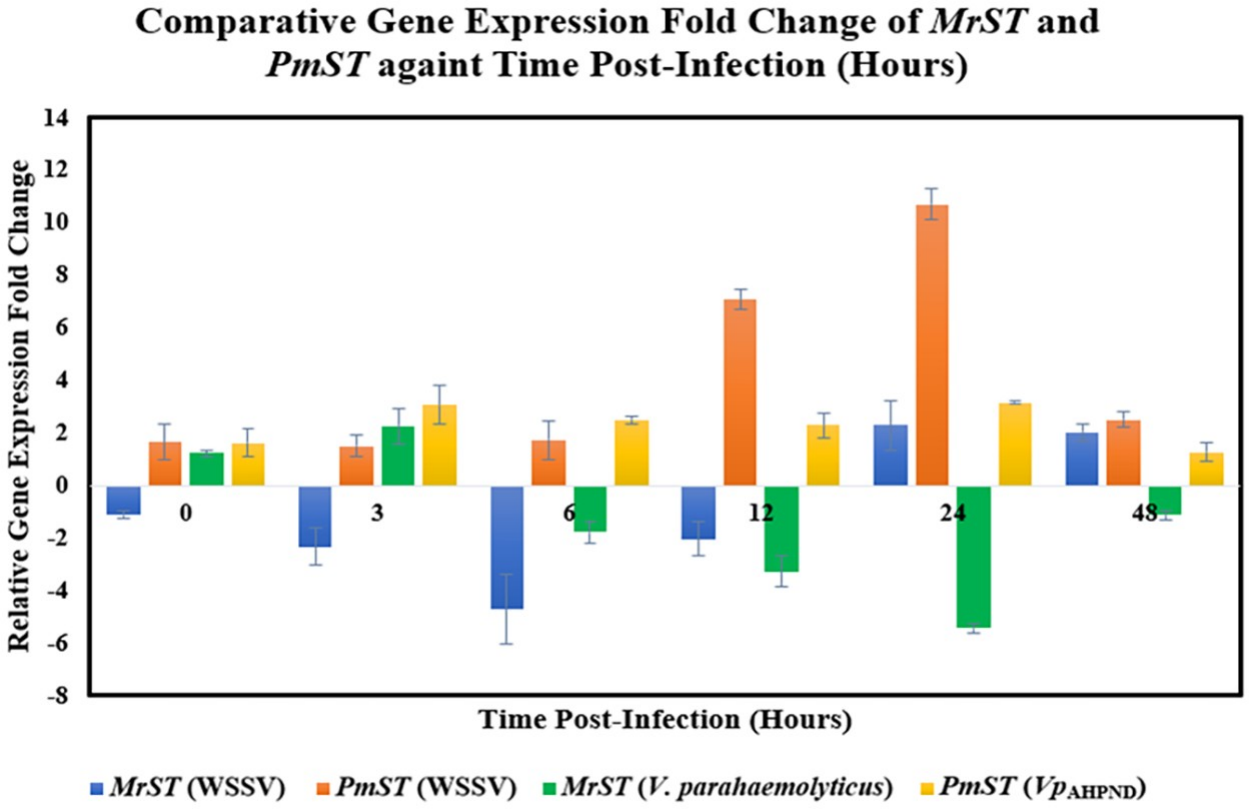
Comparative gene expression fold change of *MrST* and *PmST* against time post-infection (hours) (WSSV and *V*. *parahaemolyticus*/*Vp*_AHPND_). All relative gene expression fold changes were statistically significant (P<0.05). The error bars indicated standard deviations of the data. [*MrST* (WSSV) Fold changes: 0 hpi: -1.113; 3 hpi: -2.332; 6 hpi: -4.699; 12 hpi: -2.038; 24 hpi: 2.287; 48 hpi: 2.017]. [*PmST* (WSSV) Fold changes: 0 hpi: 1.650; 3 hpi: 1.500; 6 hpi: 1.729; 12 hpi: 7.082; 24 hpi: 10.691; 48 hpi: 2.510]. [*MrST* (*V*. *parahaemolyticus*) Fold changes: 0 hpi: 1.238; 3 hpi: 2.272; 6 hpi: -1.766; 12 hpi: -3.262; 24 hpi: -5.429; 48 hpi: -1.125]. [*PmST* (*Vp*_AHPND_) Fold changes: 0 hpi: 1.622; 3 hpi: 3.059; 6 hpi: 2.500; 12 hpi: 2.298; 24 hpi: 3.139; 48 hpi: 1.273].

The relative gene expression fold changes of *MrST* [50] and *PmST* (disease tolerant strain) across different post-infection time points of WSSV and *V*. *parahaemolyticus*/*Vp*_AHPND_ infections were determined and compared as shown in Fig 1. The individual treatment condition gene expression fold changes and statistical validations were provided in S1–S4 Figs and S3–S6 Tables.

By referring to Fig 1, during WSSV infection, *MrST* gene expressions were initially down-regulated with the greatest down-regulation detected at 6 hpi, followed by slight up-regulations at 24 and 48 hpi. Whereas *PmST* gene expressions were generally up-regulated with sharp up-regulation at 12 hpi and highest peak at 24 hpi. On the other hand, in response to bacterial (*V*. *parahaemolyticus*/*Vp*_AHPND_) infections, *MrST* gene expressions demonstrated slight up-regulation at 3 hpi followed by down-regulation from 6 hpi to 48 hpi with the lowest point at 24 hpi. *PmST* gene expressions showed significant up-regulation at 3 hpi, which was maintained until 24 hpi, and reverted to normal at 48 hpi. Intriguingly, *MrST* gene expressions were significantly down-regulated during both viral (early post-infection time points) and bacterial (later post-infection time points) infections compared to the significantly up-regulated *PmST* gene expressions.

### 3.2 Sequence comparison between MrST, PmST, and *L*. *vannamei* STAT (LvST)

**Fig 2:**
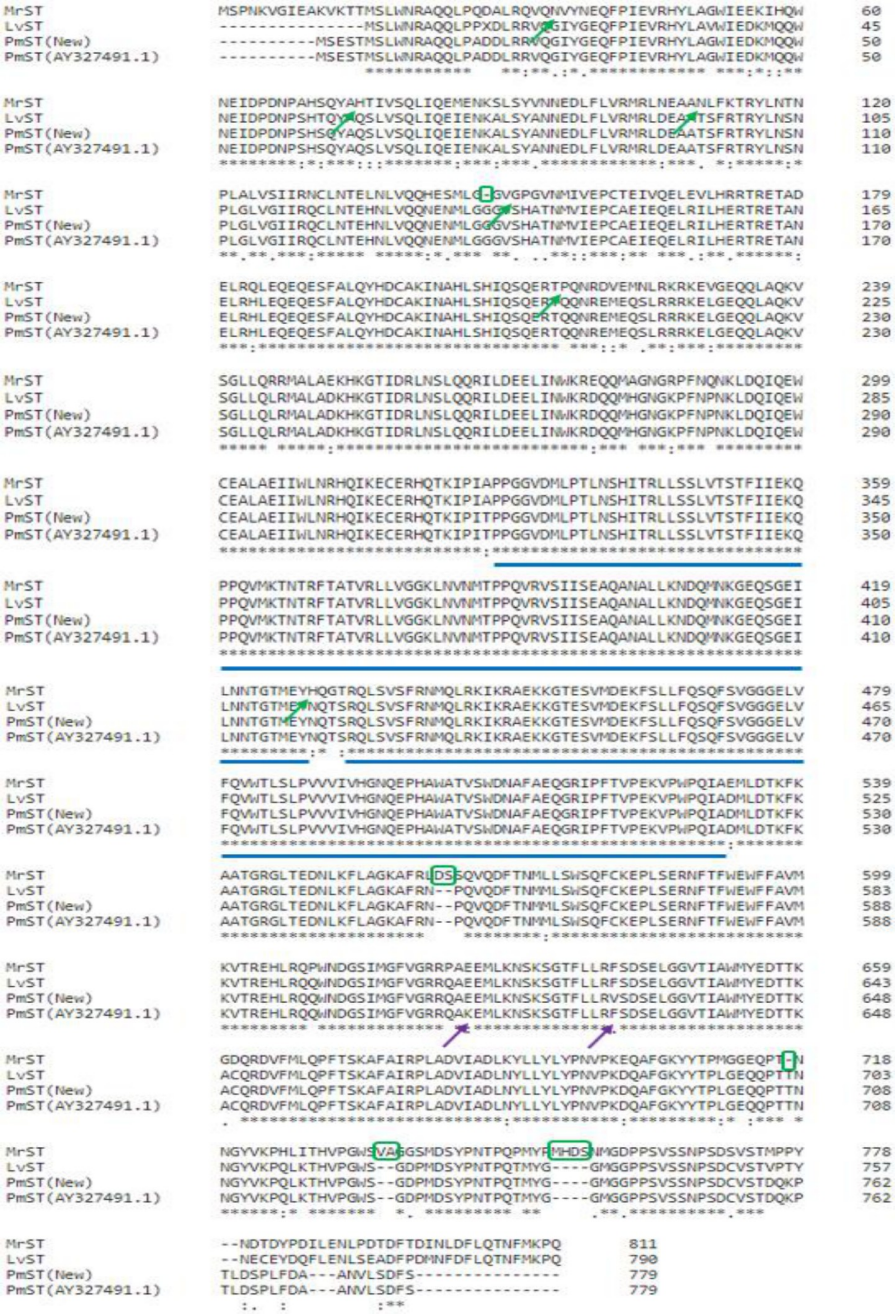
Sequence comparison of MrST, LvST, PmST (New) (disease tolerant strain), and PmST (Accession number: AY327491.1) translated amino acid sequences. * represents common conserved sites between all sequences. 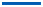 represents important long conserved overlaps between all sequences. 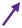 represents important divergent sites between PmST (cross-bred disease tolerant strain) and PmST (Accession number: AY327491.1). 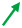 represents important divergent sites between MrST and other STAT sequences. 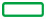 represents important amino acid addition or deletion between MrST and other STAT sequences.

Several selected shrimp STAT complete cds sequences, *MrST* (Accession Number: **KT380661**), *PmST* (disease tolerant strain), *PmST* (Accession Number: **AY327491.1**), and *LvST* (Accession Number: **HQ228176.1**) were obtained and compared using Clustal Omega software at translated amino acid (Fig 2) and nucleotide (S5 Fig) levels. The important conserved and diverged sites between the aligned sequences were marked by which a significant number of diverged sites were located at the 5’ UTR and 3’ UTR regions.

At nucleotide level, the major conserved area was found at the middle region whereas long conserved overlaps were more frequently identified at the start and end regions. Important diverged sites determined between *PmST* (disease tolerant strain) and *PmST* (**AY327491.1**) included 4, 11, 42, 179, 335, 1268, 1959, 1991, 2004, 2487, 2488, 2489, and 2491 bp. Nucleotide additions or deletions of *MrST* sequence compared to other STAT sequences were mainly identified at the regions of 16–17, 33, 56–57, 81–82, 108–110, 1786–1791, 2256–2257, and 2310–2315 bp. The major nucleotide diverged sites of *MrST* sequence compared to other STAT sequences were concentrated at the 5’ UTR, 307, 478, 730, 770, 977, 1224, 1780, 1926, 1995, 2011, 2081, 2172, 2240, 2280, and 2367 bp regions.

Besides that, at translated amino acid level, both major conserved area and long conserved overlaps between compared STAT sequences were located at the middle region. Despite the multiple important diverged sites identified between the two compared *PmST* nucleotide sequences, at amino acid level, only two amino acid alterations were discovered at positions of 614 aa [K (Lysine) to E (Glutamic Acid)] and 629 aa [F (Phenylalanine) to V (Valine)] from *PmST* (**AY327491.1**) to *PmST* (disease tolerant strain) respectively. Amino acid additions or deletions of MrST sequence compared to other STAT sequences were determined at the regions of 147–148, 562–563, 717–718, 735–736, and 753–756 aa. The major amino acid diverged sites of MrST sequence compared to other STAT sequences were located at the 35, 75, 110, 150, 215, and 429 aa regions. Interestingly, aligned *MrST* and *LvST* sequences had overlapping stop codon positions.

Homologous STAT amino acid sequences of MrST were determined through NCBI homology protein BLAST search, which included *F*. *chinensis* (89%), *Scylla paramamosain* (88%), *Eriocheir sinensis* (87%), *L*. *vannamei* (87%), *Marsupenaeus japonicus* (86%), and *Cryptotermes secundus* (69%). Whereas for PmST amino acid sequence, the homologous STAT amino acid sequences identified were *L*. *vannamei* (99%), *F*. *chinensis* (99%), *M*. *japonicus* (95%), *Portunus trituberculatus* (87%), *S*. *paramamosain* (86%), *E*. *sinensis* (85%), and *Armadillidium nasatum* (67%).

In addition, the phylogenetic analyses of MrST, PmST, and homologous STAT nucleotide and amino acid sequences were shown in S6A and S6B Fig. The *PmST* nucleotide sequence was most closely related to *F*. *chinensis* STAT, followed by *L*. *vannamei* STAT, *M*. *japonicus* STAT, and finally *MrST* nucleotide sequences within the same evolutionary clade compared to other 10 homologous STAT nucleotide sequences (S6A Fig). Whereas the PmST amino acid sequence had the closest relationship to *L*. *vannamei* STAT, followed by *F*. *chinensis* STAT, *M*. *japonicus* STAT, and *MrST* amino acid sequences within the same evolutionary clade (S6B Fig).

### 3.3 *In silico* structural comparison between MrST and PmST sequences

A full-length complete cds *PmST* (disease tolerant strain) nucleotide sequence of 2491 bp was obtained through PCR, Sanger Sequencing, sequence alignment, and trimming. *In silico* structural characterization was then conducted using the 2906 bp *MrST* (Accession Number: **KT380661)** and 2491 bp *PmST* (disease tolerant strain) nucleotide sequences.

Based on the analysis results of NCBI ORF Finder, ExPASy Translate, Show Translation, ProtParam, and NCBI CDD, *MrST* nucleotide sequence had an ORF region of 2436 bp out of the total 2906 bp (S7A Fig) encoding for a 811 aa long protein (S7B Fig) with theoretical isoelectric point of 6.04 and molecular mass of 93 kDa. On the other hand, a 2340 bp ORF region was determined from the 2491 bp *PmST* nucleotide sequence (S8A Fig) encoding for a 779 aa long protein (S8B Fig). This translated amino acid sequence had theoretical isoelectric point of 6.11 and molecular mass of 89 kDa. Four functional conserved domains were identified from the MrST and PmST protein sequences, which included STAT_int domain (protein interaction) (17 aa-141 aa; 7 aa-133 aa), STAT5_CCD domain (coiled-coil) (159 aa-352 aa; 150 aa-343 aa), STAT_bind domain (DNA binding) (354 aa-603 aa; 345 aa-592 aa), and SH2 domain (Src homology 2) (594 aa-710 aa; 583 aa-699 aa) respectively (S9A and S9B Fig).

Both MrST and PmST protein sequences were predicted to be intracellular and non-transmembrane through Protter analysis. MrST and PmST proteins had high probabilities to be located within cytoplasmic region (estimated probability of 0.61) and nuclear region (estimated probability of 0.94) respectively based on the MultiLoc 2 prediction analysis. The secondary structures of MrST and PmST protein sequences were predicted as shown in S10A and S10B Fig. For MrST protein sequence, 23 high probability common motifs and 1 Src homology 2 (SH2) domain profile (probability score, 13.148) were found (S11A Fig). Whereas 25 high probability common motifs and 1 SH2 domain profile (probability score, 13.519) were matched to PmST protein sequence (S11B Fig). 3D protein structures of MrST and PmST protein sequences were predicted which contained α-helix, β-sheet, and coil structures (S12A and S12B Fig). The optimal model template used for both structures was *Homo sapiens* STAT 5A (PDB ID: 1y1u1.A) (QMEAN: -2.04; -2.44).

### 3.4 Comparative transcriptomic differentially expressed genes (DEGs) analysis

**Fig 3:**
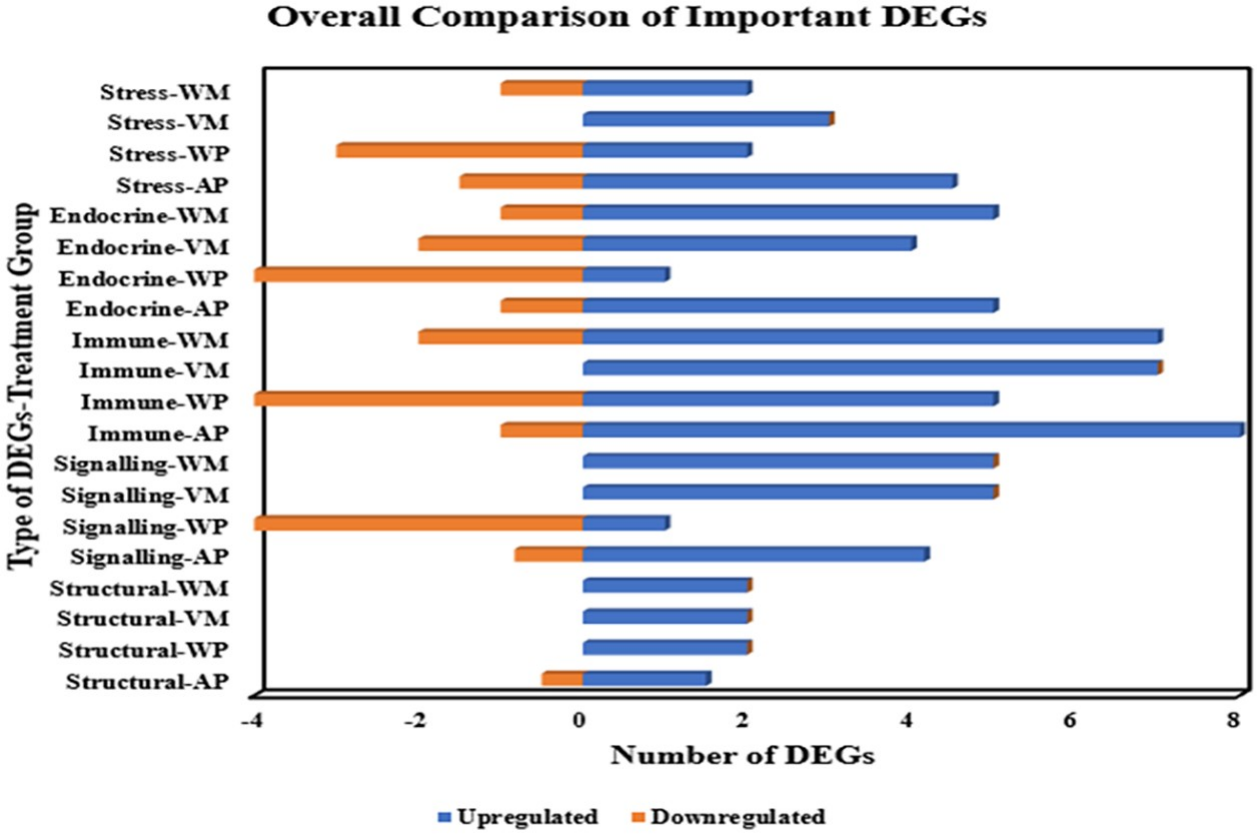
Overall comparison of important DEGs involving type of DEGs-treatment group and number of DEGs. Treatment Groups: WM: WSSV-infected *M*. *rosenbergii*; VM: *V*. *parahaemolyticus*-infected *M*. *rosenbergii*; WP: WSSV-infected *P*. *monodon* at 12 dpi; AP: *Vp*_AHPND_-infected *P*. *monodon*. Stress DEGs: Inositol 1,4,5-trisphosphate receptor, Dopamine N-acetyltransferase, Hsp90, Apoptosis-stimulating of p53 protein 1, Caspase, Apoptosis-inducing factor (AIF). Endocrine DEGs: Mitochondrial coenzyme A transporter, ATP binding cassette transmembrane transporter, Trehalose transporter, Polysaccharide lyase, Trypsin, Peroxisomal acyl-coenzyme A oxidase. Immune DEGs: Transglutaminase, C-type Lectin, HMGB, ALF1, ALF3, proPO, Superoxide Dismutase, Glutathione Peroxidase, Catalase. Signalling DEGs: Ceramide synthase, Calcium-activated chloride channel regulator, Inward rectifier potassium channel, STAT, IMD, TBK1. Structural DEGs: Actin, Ankyrin.

The important stress, endocrine, immune, signalling, and structural DEGs of *M*. *rosenbergii* and *P*. *monodon* during WSSV and *V*. *parahaemolyticus*/*Vp*_AHPND_ infections were identified and compared in the Fig 3 below. More details including gene identities, differential expression values, and annotation sources were given in the S7 Table.

Based on Fig 3, a significant higher number of up-regulated DEGs compared to down-regulated DEGs can be observed. *M*. *rosenbergii* treatment groups had more up-regulated DEGs compared to *P*. *monodon* treatment groups. WP group possessed higher number of down-regulated DEGs compared to other treatment groups. Some DEGs were only down-regulated in WP group involving inositol 1,4,5-trisphosphate receptor, apoptosis-stimulating of p53 protein 1, mitochondrial coenzyme A transporter, polysaccharide lyase, trypsin, C-type Lectin, proPO, ceramide synthase, STAT, and TBK1. Intriguingly, Hsp90, transglutaminase, ALF1, ALF3, and ankyrin demonstrated sole up-regulation pattern across all treatment groups. Peroxisomal acyl-coenzyme A oxidase and catalase were up-regulated in *M*. *rosenbergii* treatment groups while down-regulated in *P*. *monodon* treatment groups. On the other hand, trehalose transporter was down-regulated in *M*. *rosenbergii* treatment groups while up-regulated in *P*. *monodon* treatment groups. Moreover, dopamine N-acetyltransferase and HMGB only showed up-regulation pattern in *P*. *monodon* treatment groups. Apoptosis-inducing factor (AIF) and IMD were only differentially expressed in *Vp*_AHPND_-infected treatment group. Caspase was down-regulated in all treatment groups except being up-regulated in VM group.

## 4.0 Discussion

### 4.1 Differential gene expression pattern of *MrST* and *PmST* during WSSV and *V*. *parahaemolyticus*/*Vp*_AHPND_ infections

STAT gene was chosen for gene expression analyses involving different pathogenic-challenged treatment groups because of its highly diverse gene functioning in the JAK-STAT signalling pathway which possesses stress, endocrine, and immune importance [20,21,23].

Unlike the potential up-regulation of STAT gene expressions in the early WSSV post-infection time points associated with previously described hijacking mechanism adapted by WSSV for viral replication [35], the *MrST* gene expressions were down-regulated in the early WSSV post-infection time points (Fig 1) of this study. This infers that *MrST* was negatively regulated during initial WSSV infection for viral hijacking prevention. There exists possibility of miRNA involvement in such gene regulation which can be postulated based on previously identified down-regulation of human furin gene expression by miR-24 to prevent H5N1 influenza A viral spread [61]. The subsequent upregulation of *MrST* gene expressions at 24 hpi and 48 hpi matched the post-WSSV infection STAT gene expression pattern of VP28 oral-vaccinated *P*. *monodon* [62]. Based on that gene expression pattern, the continual up-regulation of *MrST* gene expressions after 48 hpi can be inferred. This suggests the significance of *MrST* gene expression and functioning in the strong immune response of *M*. *rosenbergii* activated in response to WSSV infection similar to the improved immune response of oral-vaccinated *P*. *monodon*.

Intriguingly, elevated STAT gene expressions caused by VP28 vaccination also aided in lowering viral gene expression and thus slowed down WSSV establishment in WSSV-infected *P*. *monodon* juvenile [63]. This infers a competitive relationship between host STAT gene expression and WSSV viral gene expression. Hence, the up-regulation of *PmST* gene expressions from 3 hpi to 48 hpi in response to WSSV infection (Fig 1) in this study is suggested to be the collective effect of stronger immune response from disease-resistant *P*. *monodon* and WSSV viral hijacking.

On the other hand, for *V*. *parahaemolyticus* infection, the down-regulation pattern of *MrST* gene expressions observed (Fig 1) is supported by a similar scenario of down-regulated STAT gene expressions at later hpi of *V*. *parahaemolyticus*-infected *S*. *paramamosain* [64]. The up-regulated *PmST* gene expressions identified in response to AHPND infection (Fig 1) was inferred to be caused by activated *P*. *monodon* antibacterial immune response similar to the involvement of JAK-STAT pathway in the *M*. *japonicus* antibacterial immune response [65]. Similar scenario was also reported previously for up-regulated STAT gene expressions in *V*. *anguillarum*-challenged *F*. *chinensis* shrimps [30].

### 4.2 Important conservation and divergence between STAT sequences

The vital conserved areas determined between MrST, PmST (disease tolerant strain), PmST (**AY327491.1**), and LvST sequences (nucleotide and amino acid) can be applied in cross-species conserved primer development. This is exemplified by the development of conserved primers for decapod crustaceans (including shrimps) [66] and different bear species [67] for mitochondrial genome sequencing purpose. A probable common ancestry is inferred between *MrST* and *LvST* based on their overlapping stop codon positions at nucleotide level. This is supported by another inference of a single common ancestor origination for ALF genes of all crustaceans [68]. *MrST* sequence was most diverged from other compared STAT sequences at nucleotide (S5 Fig) and amino acid (Fig 2) levels. The differential gene expressions between *MrST* and *PmST* (Fig 1) might be significantly influenced by these genetic sequence (nucleotide and amino acid) variations involving divergence, additions or deletions. The important effect of genetic variations on the gene expressions had been highlighted by previous works [69,70].

Moreover, the two amino acid changes (614 aa and 629 aa) detected between PmST sequences compared could be essential in the enhanced functioning of PmST in disease tolerant *P*. *monodon*. These amino acid changes are caused by nonsynonymous mutations. The key outcome of such amino acid changes can be inferred to be the improvement or alteration of PmST protein recognition ability or binding affinity which can be related to some previous research findings [71–73]. The divergence at the 5’ UTR and 3’ UTR regions of the aligned STAT sequences suggests the potential involvement of pre- or post-transcriptional regulatory elements found in these regions in causing differential gene expressions and alternative disease tolerance. This is validated by previously determined significant correlation between increased gene expression and adaptive evolution in the 3’ UTR and amino acid sequence [74].

By referring to the NCBI BLAST search results, although being closely related to shrimp species (*F*. *chinensis*) (89%), MrST amino acid sequence was also strongly conserved with crab species (*S*. *paramamosain* and *E*. *sinensis*) (88%; 87%). This is supported by the close evolutionary relationship between MrST and crab species (*S*. *paramamosain* and *E*. *sinensis*) in the phylogenetic analyses conducted (S6A and S6B Fig). Intriguingly, PmST amino acid sequence had high homology to *P*. *trituberculatus* (87%) in the NCBI BLAST search. This is supported by a previously determined mitochondrial DNA similarity between *P*. *monodon* and *P*. *trituberculatus* [75]. Furthermore, there was a close clustering between *Macrobrachium* prawns and Penaeidae shrimps (*P*. *monodon*, *F*. *chinensis*, and *L*. *vananmei*) in the phylogenetic analyses (S6A and S6B Fig), which suggests a similar ancestry between them.

### 4.3 *In silico* predicted MrST and PmST structural variations

The MrST protein sequence possessed slightly lower theoretical isoelectric point (pI) (6.04) compared to PmST protein sequence (6.11). The subcellular localization prediction of MrST and PmST proteins with intracellular and non-transmembrane properties was determined to be within the cytoplasmic (P = 0.61) and nuclear (P = 0.94) regions respectively. This is validated by a previously identified correlation of protein pI with subcellular localization by which cytoplasmic region contains higher number of acidic proteins compared to nuclear region [76]. Besides that, the four functional conserved domains (STAT_int, STAT5_CCD, STAT_bind, and SH2) identified for both MrST and PmST protein sequences successfully contributed to the STAT gene identity validation of these sequences. The two amino acid changes described in Section 3.2 were found within the SH2 conserved domain which has functional importance in STAT dimerization and signalling specificity [77].

### 4.4 Additional transcriptomic insight into functional DEGs

Based on the transcriptomic DEGs displayed in Fig 3 and S7 Table, further understanding was obtained for the DEGs’ functionalities particularly those related to STAT. This is important due to the insufficient number of previous studies on the direct comparison between the transcriptomic DEGs of *M*. *rosenbergii* and *P*. *monodon* under viral and bacterial infection conditions. Overall, the significantly higher number of up-regulated DEGs compared to down-regulated DEGs is postulated to be the effect of host immune response activation during pathogenic infection. In addition, the higher number of up-regulated DEGs identified in *M*. *rosenbergii* compared to *P*. *monodon* can be correlated to its stronger immune response. Such strong immune response was previously demonstrated by the ability of adult *M*. *rosenbergii* to achieve clearance of WSSV virus compared to susceptible *P*. *monodon* [78]. Intriguingly, some down-regulated DEGs were uniquely found in the WP group which resulted in its relatively higher number of down-regulated DEGs among the compared treatment groups. This is postulated to be caused by the decreased host response of survived *P*. *monodon* after successful WSSV clearance.

The functioning of stress DEGs in the early stress-induced immunoendocrine response is inferred based on both up-regulation of these DEGs in this study and previous publications [79–82]. The up-regulation of endocrine DEGs across different treatment groups is postulated to be the effect of higher energy needs for host immune response activation and post-infection cell repair. This is validated by the significance of energy balance for stress adaptation and aquatic animal tolerance highlighted in previous publications [83,84]. The activated shrimp immune response led to the up-regulation of immune and signalling DEGs. The up-regulation of structural DEGs suggests the high probability of cell and tissue structural repair across different post-infection time points. This is supported by the identification of structural DEGs (including actin-associated genes) with cytoskeleton functions in *V*. *parahaemolyticus*-infected *L*. *vannamei* [85]. All these DEGs with stress, endocrine, immune, signalling, and structural functionalities are vital in the overall host response against invading pathogens.

Moreover, the DEGs involved in overall STAT functioning through JAK-STAT pathway include inositol 1,4,5-trisphosphate receptor (associated with intracellular calcium concentration and bradykinin hormonal activation) [86,87], hsp90 (cell proliferation and protein folding) [88–90], caspase (apoptosis) [91], ATP binding cassette transmembrane transporter (cholesterol efflux) [92], C-type Lectin (pathogen recognition) [93], HMGB (inflammatory effect; DAMP) [94,95], ALF1, ALF3 (antimicrobial activity) [28,96], superoxide dismutase, glutathione peroxidase, catalase (antioxidation activity) [97–99], and TBK1 (SOCS-mediated degradation) [100].

Besides that, other DEGs possibly or indirectly associated with JAK-STAT signalling are apoptosis-stimulating of p53 protein 1 (apoptosis) [101–103], apoptosis-inducing factor (AIF) (apoptosis) [104,105], trehalose transporter (cell proliferation) [106], trypsin (viral immune response) [107], peroxisomal acyl-coenzyme A oxidase (associated with leptin and adiponectin; apoptosis) [108,109], transglutaminase (clotting reaction during viral infection) [110], proPO (proPO activation system and melanization) [110], calcium-activated chloride channel regulator (STAT regulation of mucus production) [111,112], and IMD (another important signalling pathway component of invertebrate immune response) [113]. The remaining DEGs may have functional relevance to JAK-STAT signalling pathway, but further validations are needed due to the lack of previous studies.

Interestingly, the uniquely up-regulated DEGs, including peroxisomal acyl-coenzyme A oxidase, catalase, and TBK1 in *M*. *rosenbergii* infers their greater importance in *M*. *rosenbergii* host response. On the other hand, dopamine N-acetyltransferase, trehalose transporter, and HMGB showed unique up-regulation in *P*. *monodon* during pathogenic infections, which suggests their stronger importance in *P*. *monodon* host response as well. The special functional importance of AIF and IMD signalling pathway in AP group may be further investigated to gain deeper understanding into their sole differential expressions in AP group.

Overall, a synergistic functioning of shrimp stress, endocrine, immune, signalling, and structural genes during pathogenic infections can be postulated which is vital for host survival and elimination of invading pathogens. Moreover, these DEGs can be jointly proposed as Survival Adaptation Molecular Patterns (SAMPs) with STAT as one of the crucial signalling components.

## 5.0 Conclusions

In conclusion, during WSSV and *V*. *parahaemolyticus*/*Vp*_AHPND_ infections, *MrST* gene expressions were significantly down-regulated (during either early or later post-infection time points) whereas *PmST* gene expressions were only significantly up-regulated. In addition, the sequence and structural comparison of MrST and PmST provided significant insight into the important similarities or differences between the compared shrimp STAT sequences. These differences were inferred to be one of the deciding factors resulting in the differential gene expression patterns observed. STAT gene plays vital diverse roles in JAK-STAT signalling pathway especially during pathogenic infections. Hence, the systematic comparison of selected omics data was done to identify the important DEGs (stress, endocrine, immune, signalling, and structural) in *M*. *rosenbergii* and *P*. *monodon* when exposed to WSSV and *V*. *parahaemolyticus*/*Vp*_AHPND_ infections focusing on those involved in STAT functioning or potentially associated with JAK-STAT signalling. The functional grouping of these DEGs validated the diverse signalling roles of STAT.

Overall, the findings of this study will be able to provide valuable insight for future research towards better understanding of the shrimp immune response especially STAT gene functioning. This study also possesses novelty in the emphasis of the stress and endocrine DEGs because these DEGs are easily neglected normally with more research focus given to immune DEGs. However, these DEGs are important as well because stress DEGs function as alert and trigger factors whereas endocrine DEGs function as regulatory and survival factors. The qPCR primers designed, sequence and structural divergence identified, and important DEGs obtained can be applied for shrimp health or immune response activation diagnostic purpose. STAT gene can also be proposed as a suitable candidate for the study of immune response enhancement or regulation due to its diverse signalling importance in shrimp immunity and survival.

## Supporting information

S1 Fig Relative gene expression fold change of *MrST* in response to WSSV infection. *All relative gene expression fold changes were statistically significant (P<0.05). *a, b, and c represent different subsets obtained in Post Hoc Duncan Test. *The error bars indicated standard deviations of the data. [*MrST* (WSSV) Fold changes: 0 hpi: -1.113; 3 hpi: -2.332; 6 hpi: -4.699; 12 hpi: -2.038; 24 hpi: 2.287; 48 hpi: 2.017].
(DOCX)

S2 Fig Relative gene expression fold change of *MrST* in response to *V*. *parahaemolyticus* infection. *All relative gene expression fold changes were statistically significant (P<0.05). *a, b, c, and d represent different subsets obtained in Post Hoc Duncan Test. *The error bars indicated standard deviations of the data. [*MrST* (*V*. *parahaemolyticus*) Fold changes: 0 hpi: 1.238; 3 hpi: 2.272; 6 hpi: -1.766; 12 hpi: -3.262; 24 hpi: -5.429; 48 hpi: -1.125].
(DOCX)

S3 Fig Relative gene expression fold change of *PmST* in response to WSSV infection. *All relative gene expression fold changes were statistically significant (P<0.05). *a, b, and c represent different subsets obtained in Post Hoc Duncan Test. *The error bars indicated standard deviations of the data. [*PmST* (WSSV) Fold changes: 0 hpi: 1.650; 3 hpi: 1.500; 6 hpi: 1.729; 12 hpi: 7.082; 24 hpi: 10.691; 48 hpi: 2.510].
(DOCX)

S4 Fig Relative gene expression fold change of *PmST* in response to *Vp*_AHPND_ infection. *All relative gene expression fold changes were statistically significant (P<0.05). *a, b, and c represent different subsets obtained in Post Hoc Duncan Test. *The error bars indicated standard deviations of the data. [*PmST* (*Vp*_AHPND_) Fold changes: 0 hpi: 1.622; 3 hpi: 3.059; 6 hpi: 2.500; 12 hpi: 2.298; 24 hpi: 3.139; 48 hpi: 1.273].
(DOCX)

S5 FigSequence comparison of *MrST*, *LvST*, *PmST* (cross-bred disease tolerant strain) [labelled as PmST(New)], and *PmST* (Accession number: AY327491.1) STAT nucleotide sequences using Clustal Omega software.* represents common conserved sites between all sequences. 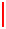 represents the start and stop codons of the ORF regions. 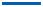 represents important long conserved overlaps between all sequences. 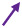 represents important divergent sites between *PmST* (cross-bred disease tolerant strain) and *PmST* (Accession number: AY327491.1). 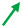 represents important divergent sites between *MrST* and other STAT sequences. 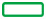 represents important nucleotide addition or deletion between *MrST* and other STAT sequences.(DOCX)

S6 Fig Phylogenetic comparison of *M*. *rosenbergii* STAT (MrST) and *P*. *monodon* STAT (PmST) (disease tolerant strain and AY327491.1) sequences with 13 homologous sequences retrieved from NCBI database using MEGA 7 software through Maximum Likelihood method with bootstrap value of 1000. A) Phylogenetic tree generated from *M*. *rosenbergii* STAT (*MrST*), *P*. *monodon* STAT (*PmST*) (disease tolerant strain and AY327491.1), and other homologous STAT nucleotide sequences (Tamura-Nei model). B) Phylogenetic tree generated from *M*. *rosenbergii* STAT (MrST), *P*. *monodon* STAT (PmST) (cross-bred disease tolerant strain and AY327491.1), and other homologous STAT amino acid sequences (Jones-Taylor-Thornton (JTT) model). *PmST (disease tolerant strain) nucleotide and amino acid sequences were labelled as “New”.
(DOCX)

S7 Fig Nucleotide and translated amino acid sequences of *M*. *rosenbergii* STAT (MrST). A) Nucleotide sequence of *M*. *rosenbergii* STAT (*MrST*) obtained (full coding sequence). A total of 2906 base pair (bp) were observed for *MrST*, with an ORF of 2436 bp (ORF labelled in blue). B) MrST amino acid sequence numbered from N-terminus aligned with respective ORF nucleotide sequence. The protein consists of 811 amino acids (amino acid labelled in blue).
(DOCX)

S8 Fig Nucleotide and translated amino acid sequences of *P*. *monodon* STAT (PmST). A) Nucleotide sequence of *Penaeus monodon* STAT (*PmST*) obtained (full coding sequence). A total of 2491 base pair (bp) were observed for *PmST*, with an ORF of 2340 bp (ORF labelled in blue). B) PmST amino acid sequence numbered from N-terminus aligned with respective ORF nucleotide sequence. The protein consists of 779 amino acids (amino acid labelled in blue).
(DOCX)

S9 Fig NCBI Conserved Domain Search of *M*. *rosenbergii* STAT (MrST) and *P*. *monodon* STAT (PmST). A) NCBI Conserved Domain Search of MrST protein sequence demonstrating four functional domains, namely STAT_int (17 aa-141 aa), STAT5_CCD (159 aa-352 aa), STAT_bind (354 aa-603 aa), and SH2_STAT (594 aa-710 aa). B) NCBI Conserved Domain Search of PmST protein sequence demonstrating four functional domains, namely STAT_int (7 aa-133 aa), STAT5_CCD (150 aa-343 aa), STAT_bind (345 aa-592 aa), and SH2_STAT (583 aa-699 aa).
(DOCX)

S10 Fig Secondary Structure Prediction of *M*. *rosenbergii* STAT (MrST) and *P*. *monodon* STAT (PmST) (Cross-bred Disease Tolerant Strain) Protein Sequences using PSIPRED. A) Predicted secondary structure of *M*. *rosenbergii* STAT (MrST) protein sequence. B) Predicted secondary structure of *P*. *monodon* STAT (PmST) protein sequence.
(DOCX)

S11 Fig Protein Domains, Families, and Functional Sites Prediction of *M*. *rosenbergii* STAT (MrST) and *P*. *monodon* STAT (PmST) Protein Sequences using PROSITE. A) Predicted domains, families, and functional sites of *M*. *rosenbergii* STAT (MrST) protein sequence. B) Predicted domains, families, and functional sites of *P*. *monodon* STAT (PmST) protein sequence.
(DOCX)

S12 Fig 3D Protein Structure Prediction of *M*. *rosenbergii* STAT (MrST) and *P*. *monodon* STAT (PmST) Protein Sequences using SWISS-MODEL. A) Predicted 3D protein structure of *M*. *rosenbergii* STAT (MrST) protein sequence. B) Predicted 3D protein structure of *P*. *monodon* STAT (PmST) protein sequence.
(DOCX)

S1 Table The list of gene, primer pair, and probe sequences used in the qPCR experiments. (DOCX)

S2 Table The list of gene and primer pair sequences used in the PCR experiments. (DOCX)

S3 Table Statistical significance validation of *MrST* relative gene expression in response to WSSV infection using (A) One-Way ANOVA and (B) Post Hoc Duncan Test. (DOCX)

S4 Table Statistical significance validation of *MrST* relative gene expression in response to *V*. *parahaemolyticus* infection using (A) One-Way ANOVA and (B) Post Hoc Duncan Test. (DOCX)

S5 Table Statistical significance validation of *PmST* relative gene expression in response to WSSV infection using (A) One-Way ANOVA and (B) Post Hoc Duncan Test. (DOCX)

S6 Table Statistical significance validation of *PmST* relative gene expression in response to *Vp*_AHPND_ infection using (A) One-Way ANOVA and (B) Post Hoc Duncan Test. (DOCX)

S7 Table Detailed comparison of important transcriptomic DEGs identified from WSSV and *V*. *parahaemolyticus*/*Vp*_AHPND_ -infected *M*. *rosenbergii* and *P*. *monodon* samples. (DOCX)

S1 Data Raw data of qPCR analysis. (ZIP)

S1 Graphical Abstract (PDF)
