# Supplementary figures and images for: Differential STAT gene expressions of Penaeus monodon and Macrobrachium rosenbergii in response to white spot syndrome virus (WSSV) and bacterial infections: Additional insight into genetic variations and transcriptomic highlights
Source: PLoS One. 2021 Oct 15;16(10):e0258655. doi: 10.1371/journal.pone.0258655 (PMC8519450; doi:10.1371/journal.pone.0258655)

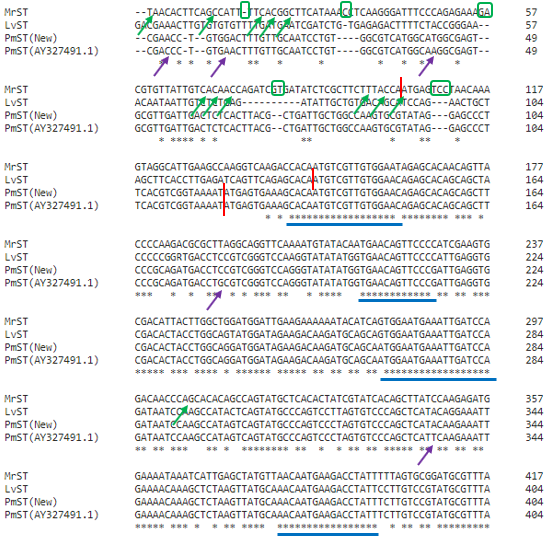


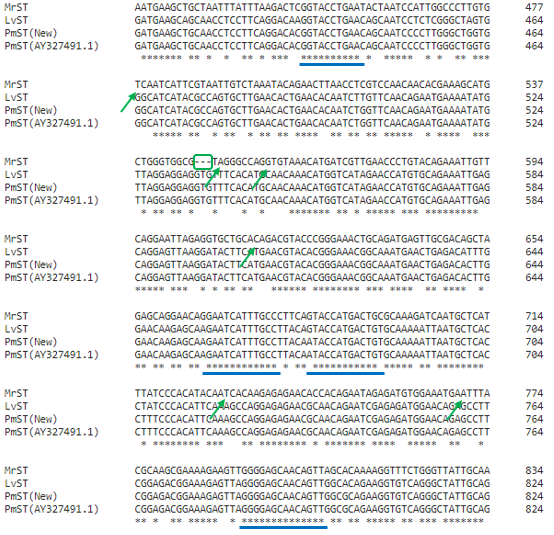


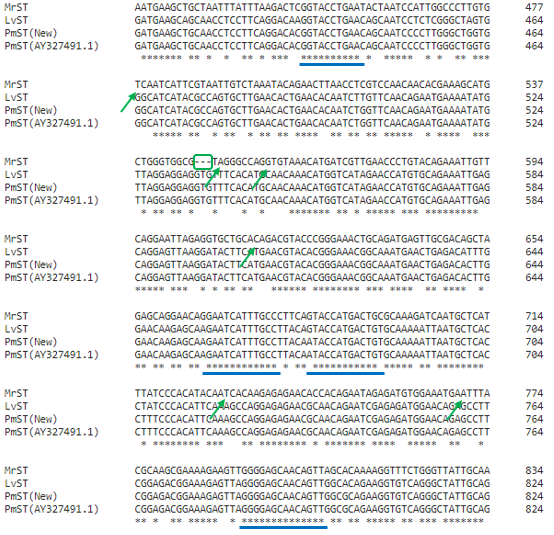


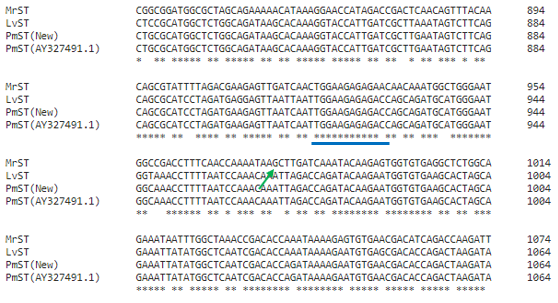


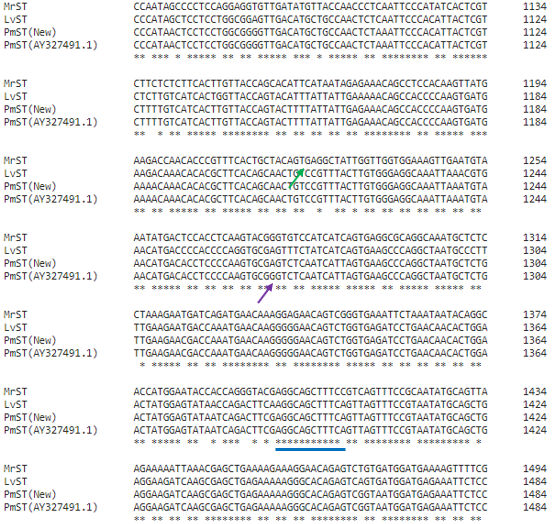


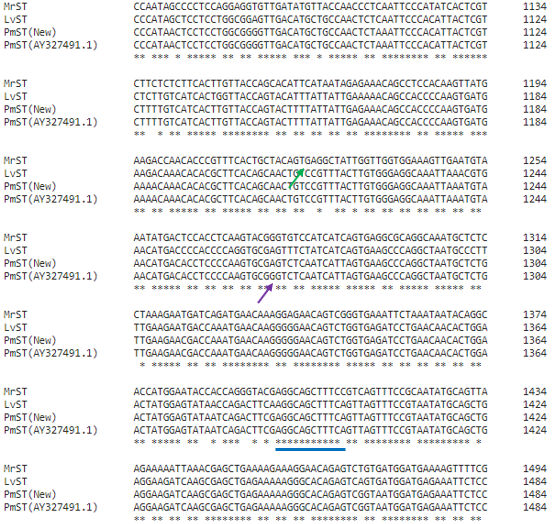


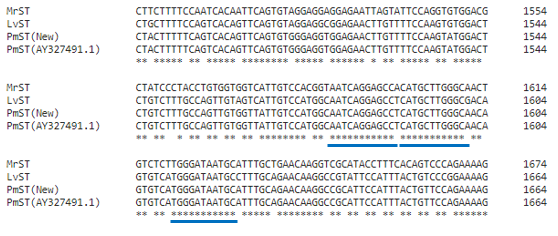


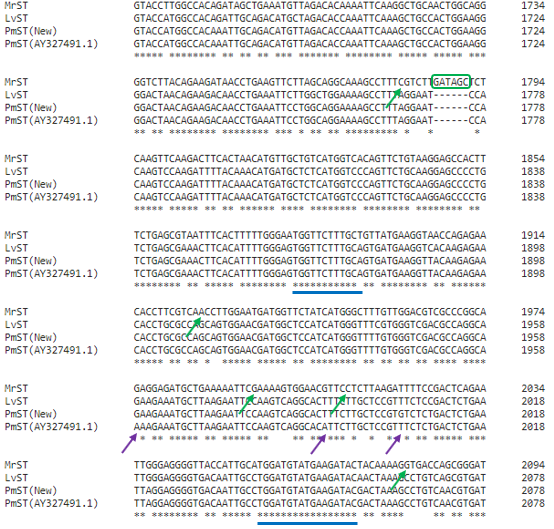


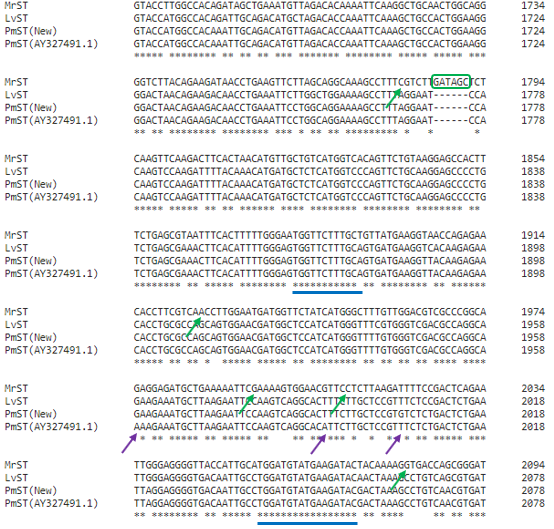

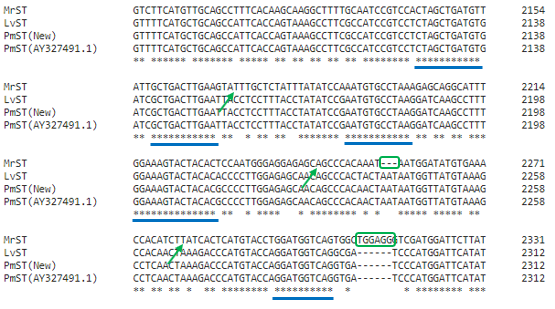


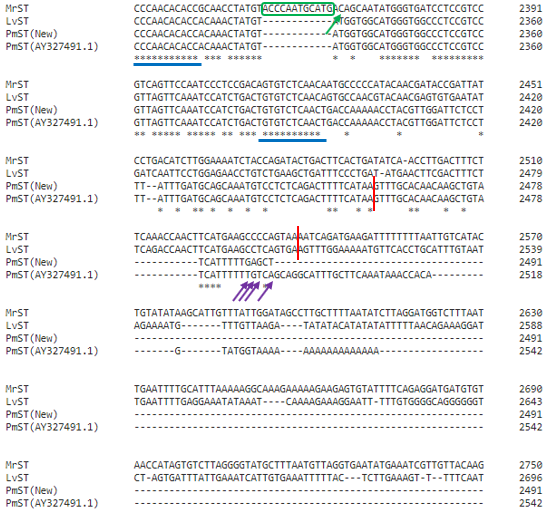


**S5 Fig**

Supplement: S5 Fig — * represents common conserved sites between all sequences. represents the start and stop codons of the ORF regions. represents important long conserved overlaps between all sequences. represents important divergent sites between PmST (cross-bred disease tolerant strain) and PmST (Accession number: AY327491.1). represents important divergent sites between MrST and other STAT sequences. represents important nucleotide addition or deletion between MrST and other STAT sequences. (DOCX) [file pone.0258655.s005.docx]

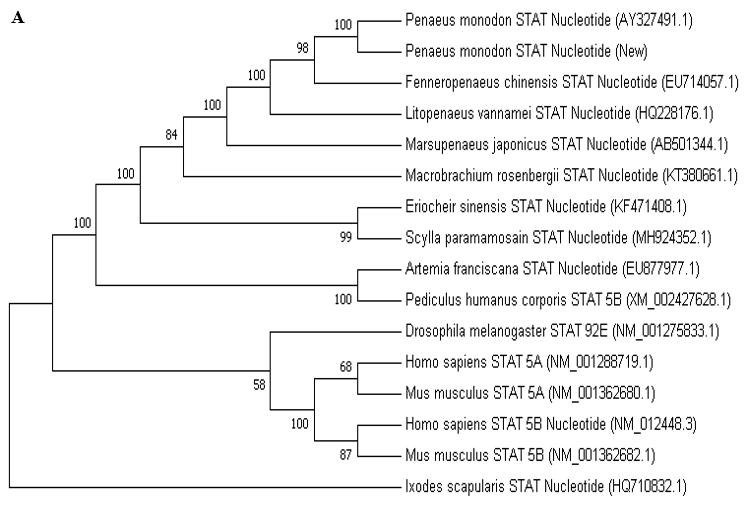


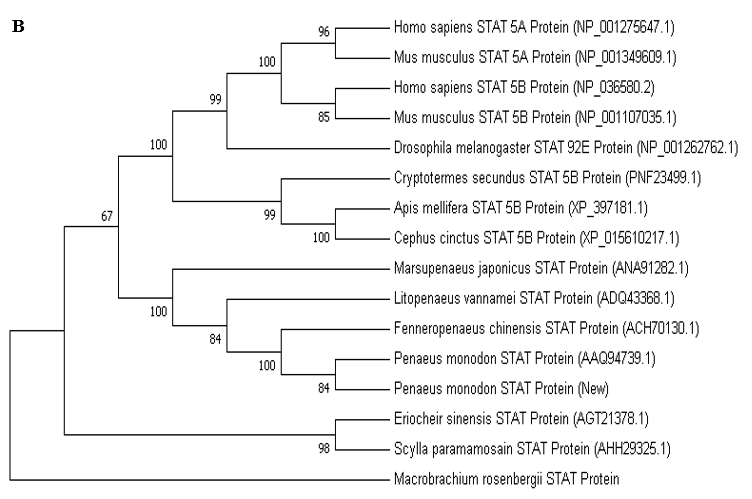


**S6 Fig**

Supplement: S6 Fig — A) Phylogenetic tree generated from M. rosenbergii STAT (MrST), P. monodon STAT (PmST) (disease tolerant strain and AY327491.1), and other homologous STAT nucleotide sequences (Tamura-Nei model). B) Phylogenetic tree generated from M. rosenbergii STAT (MrST), P. monodon STAT (PmST) (cross-bred disease tolerant strain and AY327491.1), and other homologous STAT amino acid sequences (Jones-Taylor-Thornton (JTT) model). *PmST (disease tolerant strain) nucleotide and amino acid sequences were labelled as “New”. (DOCX) [file pone.0258655.s006.docx]

**S9A Fig**


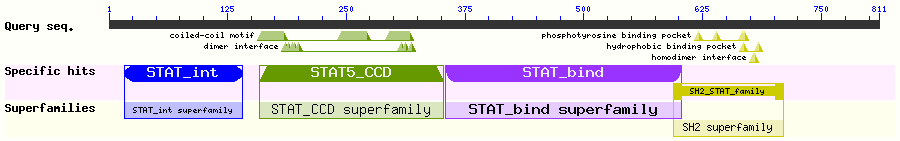


**S9B Fig**


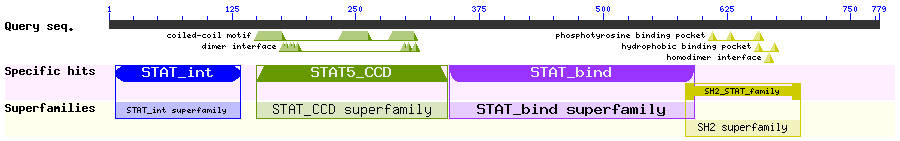


**S9 Fig**

Supplement: S9 Fig — A) NCBI Conserved Domain Search of MrST protein sequence demonstrating four functional domains, namely STAT_int (17 aa-141 aa), STAT5_CCD (159 aa-352 aa), STAT_bind (354 aa-603 aa), and SH2_STAT (594 aa-710 aa). B) NCBI Conserved Domain Search of PmST protein sequence demonstrating four functional domains, namely STAT_int (7 aa-133 aa), STAT5_CCD (150 aa-343 aa), STAT_bind (345 aa-592 aa), and SH2_STAT (583 aa-699 aa). (DOCX) [file pone.0258655.s009.docx]

**S10A Fig**


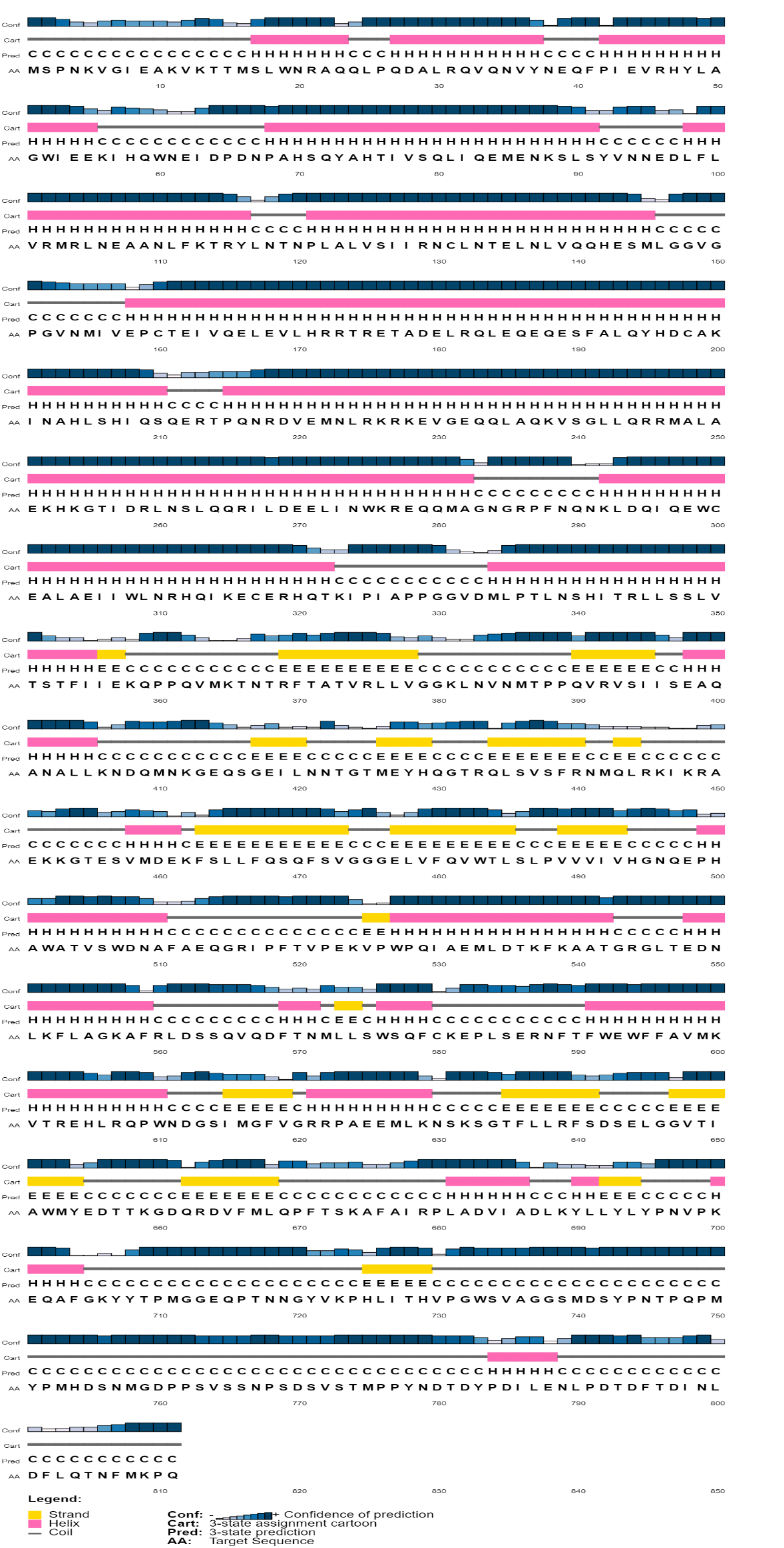


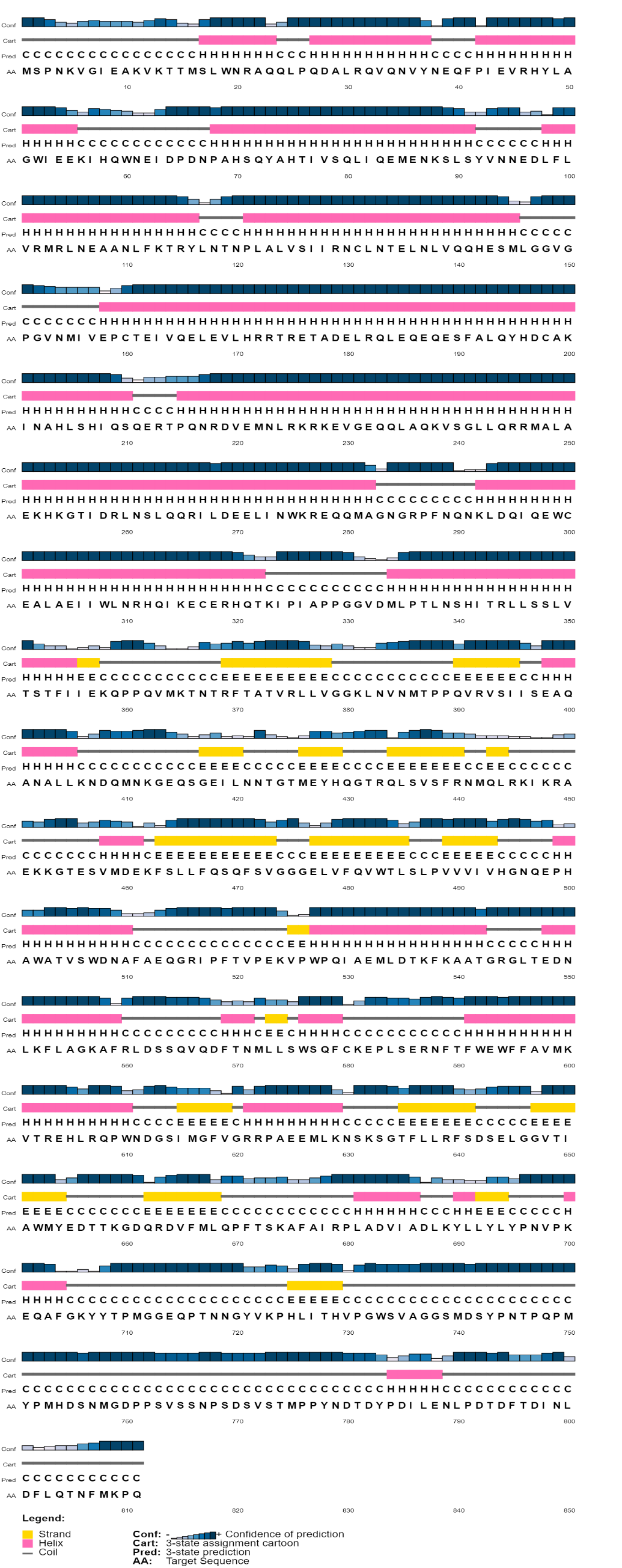


**S10B Fig**

**
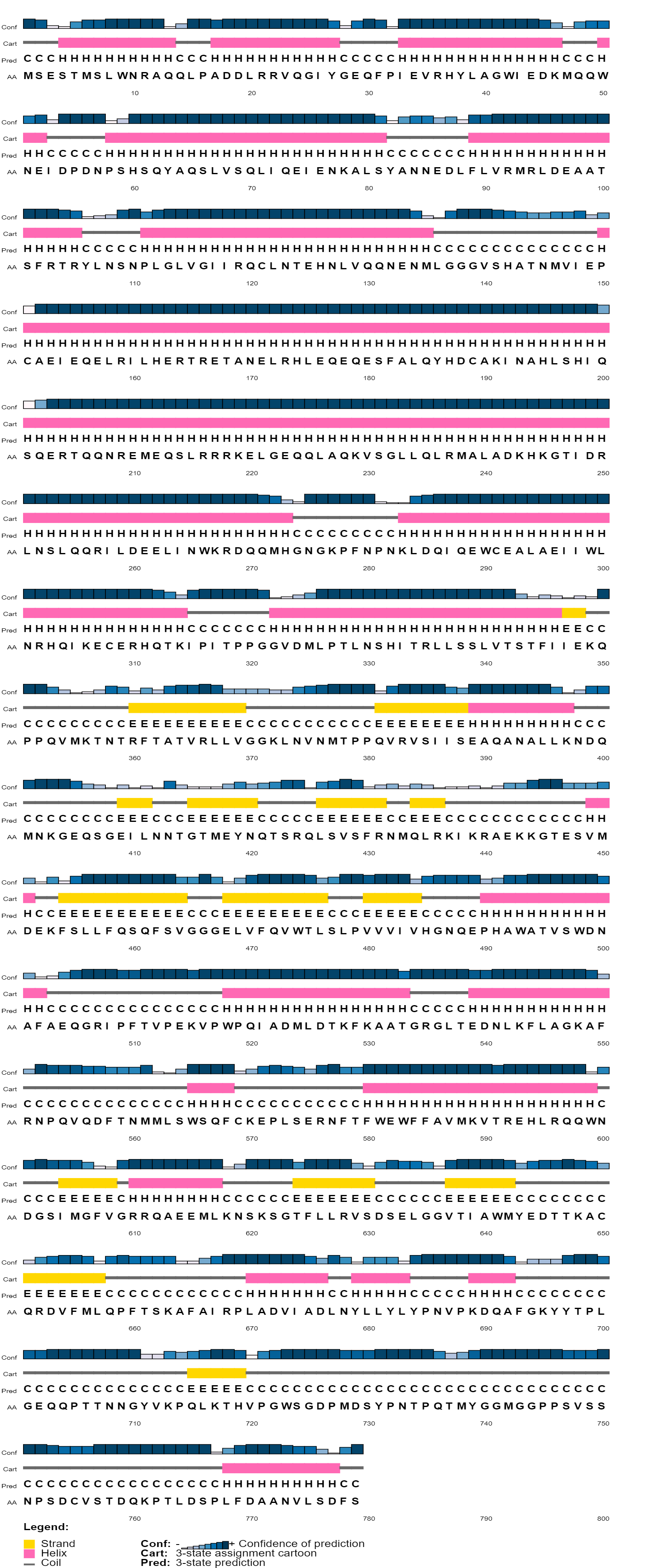
**

**
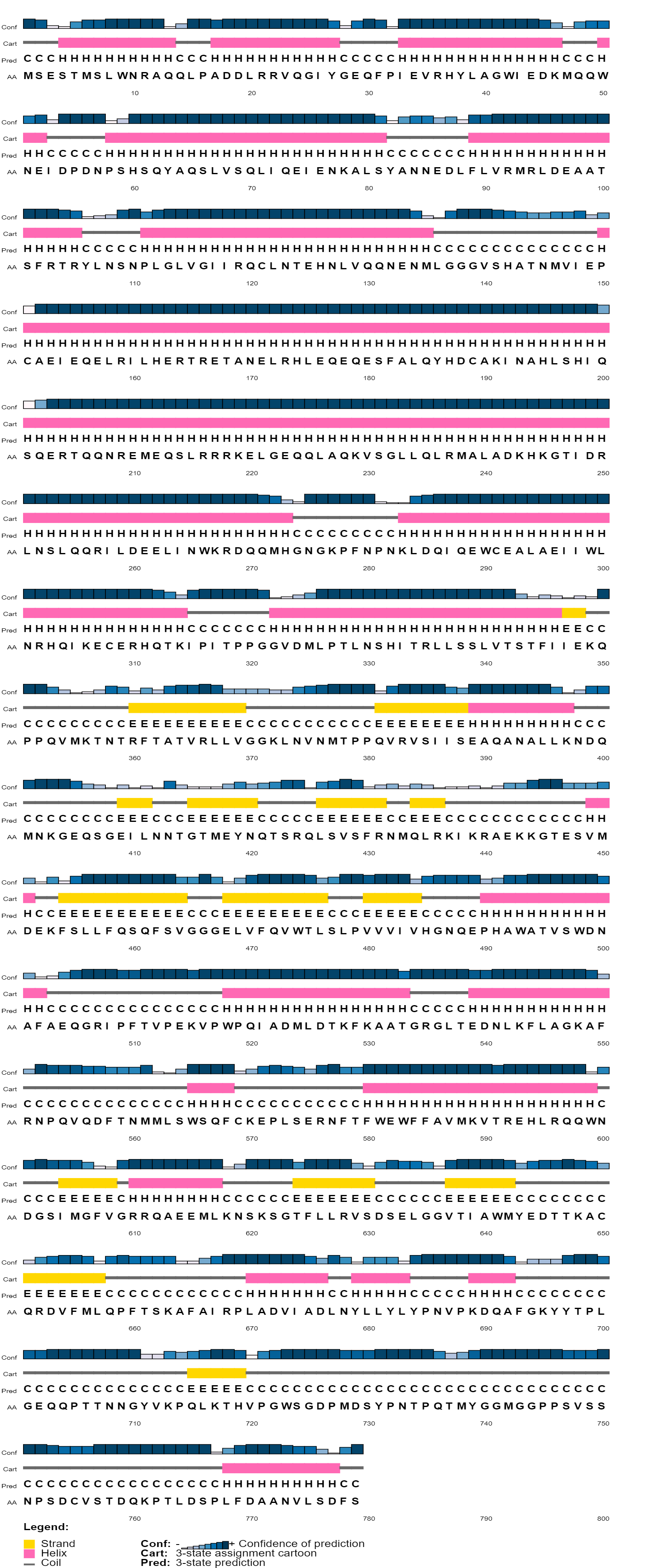
**

**S10 Fig**

Supplement: S10 Fig — A) Predicted secondary structure of M. rosenbergii STAT (MrST) protein sequence. B) Predicted secondary structure of P. monodon STAT (PmST) protein sequence. (DOCX) [file pone.0258655.s010.docx]

**S11A Fig**


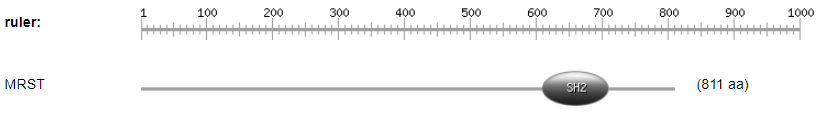


**S11B Fig**


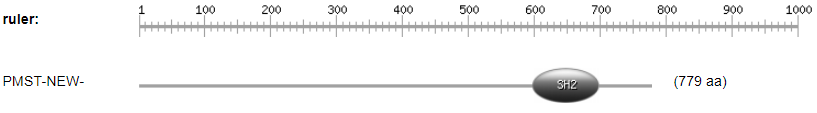


**S11 Fig**

Supplement: S11 Fig — A) Predicted domains, families, and functional sites of M. rosenbergii STAT (MrST) protein sequence. B) Predicted domains, families, and functional sites of P. monodon STAT (PmST) protein sequence. (DOCX) [file pone.0258655.s011.docx]

**S12A Fig**

**
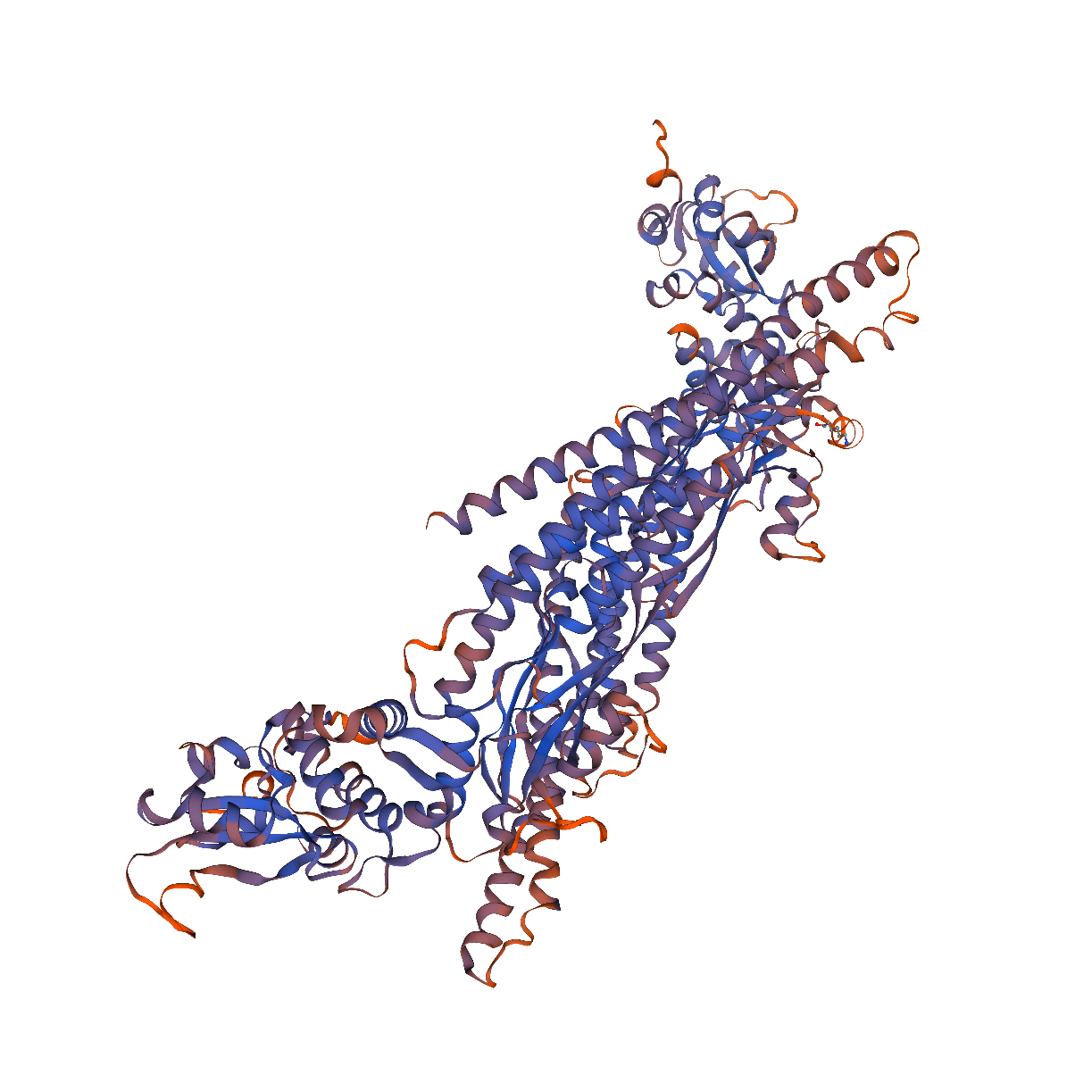
**

**α-Helix**

**β-Sheet**

**Coil**

**S12B Fig**

**
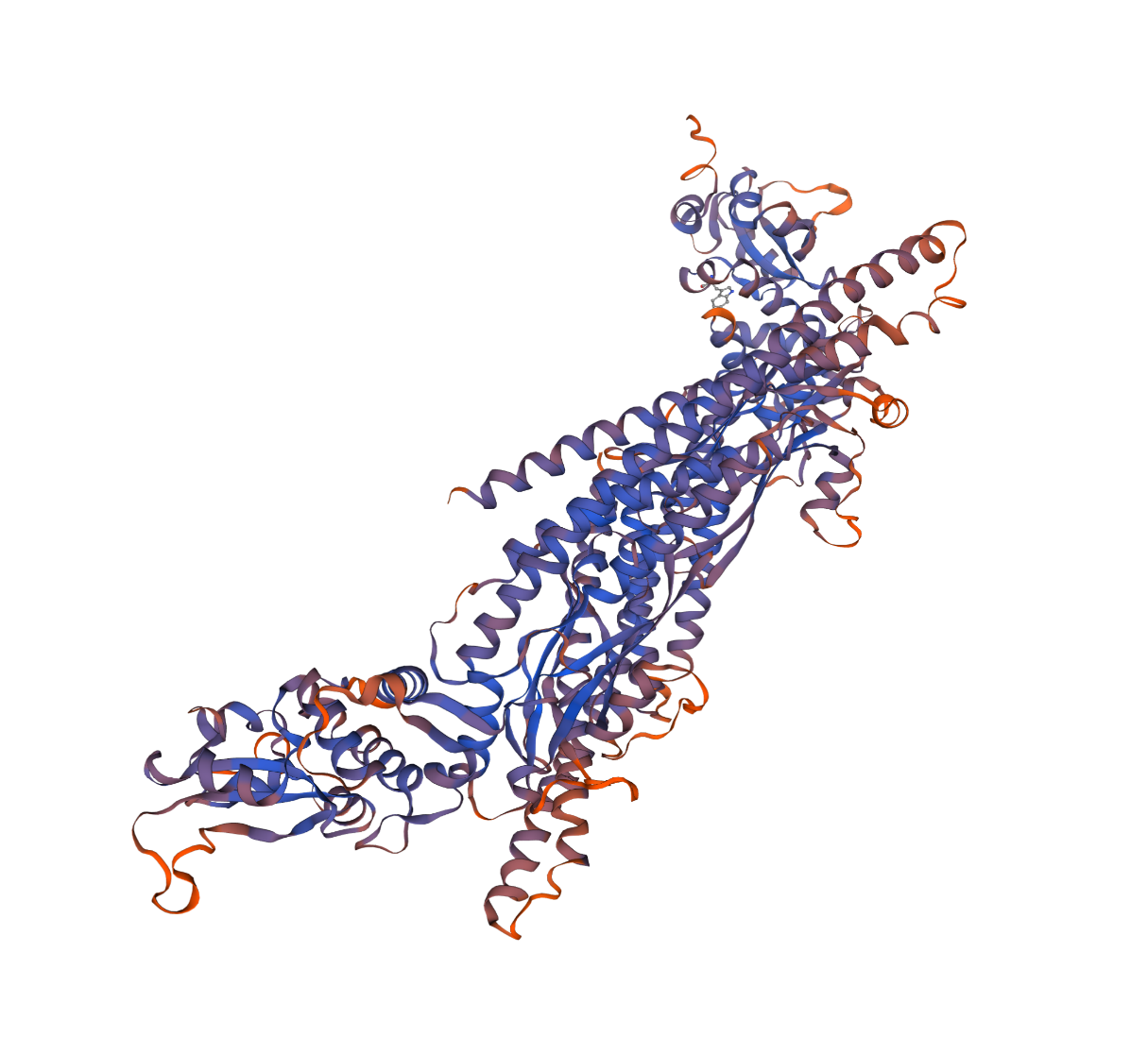
**

**β-Sheet**

**α-Helix**

**Coil**

**S12 Fig**

Supplement: S12 Fig — A) Predicted 3D protein structure of M. rosenbergii STAT (MrST) protein sequence. B) Predicted 3D protein structure of P. monodon STAT (PmST) protein sequence. (DOCX) [file pone.0258655.s012.docx]

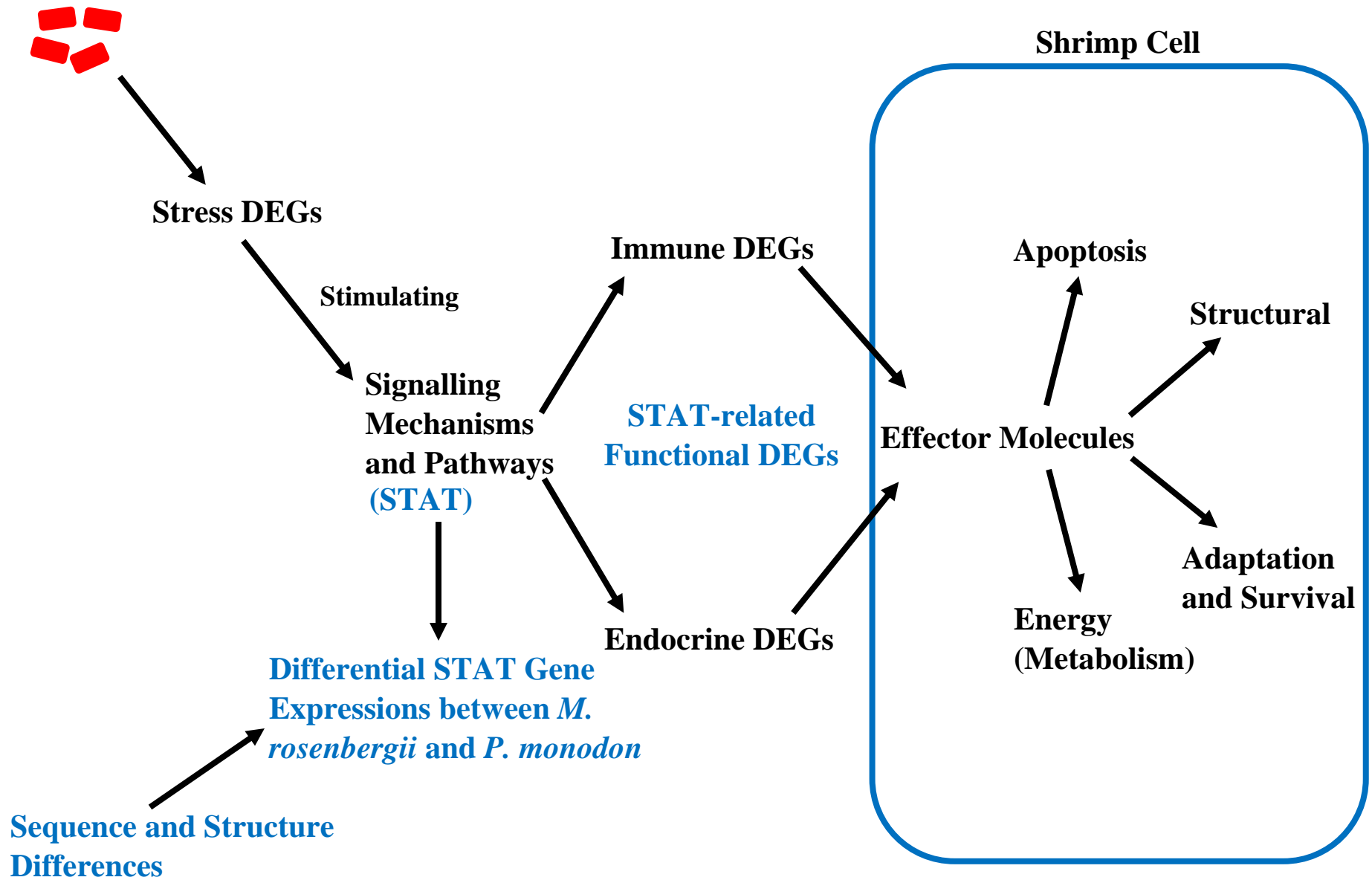

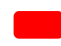 **Invading Pathogen (WSSV or *V. parahaemolyticus*/*Vp*<sub>AHPND</sub>)**

Supplement: S1 Graphical Abstract — (PDF) [file pone.0258655.s021.pdf]
