# Supplementary material for: Differential STAT gene expressions of Penaeus monodon and Macrobrachium rosenbergii in response to white spot syndrome virus (WSSV) and bacterial infections: Additional insight into genetic variations and transcriptomic highlights
Source: PLoS One. 2021 Oct 15;16(10):e0258655. doi: 10.1371/journal.pone.0258655 (PMC8519450; doi:10.1371/journal.pone.0258655)
Supplement: S7 Fig — A) Nucleotide sequence of M. rosenbergii STAT (MrST) obtained (full coding sequence). A total of 2906 base pair (bp) were observed for MrST, with an ORF of 2436 bp (ORF labelled in blue). B) MrST amino acid sequence numbered from N-terminus aligned with respective ORF nucleotide sequence. The protein consists of 811 amino acids (amino acid labelled in blue). (DOCX) [file pone.0258655.s007.docx]

**S7A Fig**

1 TAACACTTCA GCCATTTTCA CGGCTTCATA AACCTCAAGG GATTTCCCAG AGAAAGACGT 60

61 GTTATTGTCA CAACCAGATC GTGATATCTC GCTTCTTTAC CAATGAGTCC TAACAAAGTA 120

121 GGCATTGAAG CCAAGGTCAA GACCACAATG TCGTTGTGGA ATAGAGCACA ACAGTTACCC 180

181 CAAGACGCGC TTAGGCAGGT TCAAAATGTA TACAATGAAC AGTTCCCCAT CGAAGTGCGA 240

241 CATTACTTGG CTGGATGGAT TGAAGAAAAA ATACATCAGT GGAATGAAAT TGATCCAGAC 300

301 AACCCAGCAC ACAGCCAGTA TGCTCACACT ATCGTATCAC AGCTTATCCA AGAGATGGAA 360

361 AATAAATCAT TGAGCTATGT TAACAATGAA GACCTATTTT TAGTGCGGAT GCGTTTAAAT 420

421 GAAGCTGCTA ATTTATTTAA GACTCGGTAC CTGAATACTA ATCCATTGGC CCTTGTGTCA 480

481 ATCATTCGTA ATTGTCTAAA TACAGAACTT AACCTCGTCC AACAACACGA AAGCATGCTG 540

541 GGTGGCGTAG GGCCAGGTGT AAACATGATC GTTGAACCCT GTACAGAAAT TGTTCAGGAA 600

601 TTAGAGGTGC TGCACAGACG TACCCGGGAA ACTGCAGATG AGTTGCGACA GCTAGAGCAG 660

661 GAACAGGAAT CATTTGCCCT TCAGTACCAT GACTGCGCAA AGATCAATGC TCATTTATCC 720

721 CACATACAAT CACAAGAGAG AACACCACAG AATAGAGATG TGGAAATGAA TTTACGCAAG 780

781 CGAAAAGAAG TTGGGGAGCA ACAGTTAGCA CAAAAGGTTT CTGGGTTATT GCAACGGCGG 840

841 ATGGCGCTAG CAGAAAAACA TAAAGGAACC ATAGACCGAC TCAACAGTTT ACAACAGCGT 900

901 ATTTTAGACG AAGAGTTGAT CAACTGGAAG AGAGAACAAC AAATGGCTGG GAATGGCCGA 960

961 CCTTTCAACC AAAATAAGCT TGATCAAATA CAAGAGTGGT GTGAGGCTCT GGCAGAAATA 1020

1021 ATTTGGCTAA ACCGACACCA AATAAAAGAG TGTGAACGAC ATCAGACCAA GATTCCAATA 1080

1081 GCCCCTCCAG GAGGTGTTGA TATGTTACCA ACCCTCAATT CCCATATCAC TCGTCTTCTC 1140

1141 TCTTCACTTG TTACCAGCAC ATTCATAATA GAGAAACAGC CTCCACAAGT TATGAAGACC 1200

1201 AACACCCGTT TCACTGCTAC AGTGAGGCTA TTGGTTGGTG GAAAGTTGAA TGTAAATATG 1260

1261 ACTCCACCTC AAGTACGGGT GTCCATCATC AGTGAGGCGC AGGCAAATGC TCTCCTAAAG 1320

1321 AATGATCAGA TGAACAAAGG AGAACAGTCG GGTGAAATTC TAAATAATAC AGGCACCATG 1380

1381 GAATACCACC AGGGTACGAG GCAGCTTTCC GTCAGTTTCC GCAATATGCA GTTAAGAAAA 1440

1441 ATTAAACGAG CTGAAAAGAA AGGAACAGAG TCTGTGATGG ATGAAAAGTT TTCGCTTCTT 1500

1501 TTCCAATCAC AATTCAGTGT AGGAGGAGGA GAATTAGTAT TCCAGGTGTG GACGCTATCC 1560

1561 CTACCTGTGG TGGTCATTGT CCACGGTAAT CAGGAGCCAC ATGCTTGGGC AACTGTCTCT 1620

1621 TGGGATAATG CATTTGCTGA ACAAGGTCGC ATACCTTTCA CAGTCCCAGA AAAGGTACCT 1680

1681 TGGCCACAGA TAGCTGAAAT GTTAGACACA AAATTCAAGG CTGCAACTGG CAGGGGTCTT 1740

1741 ACAGAAGATA ACCTGAAGTT CTTAGCAGGC AAAGCCTTTC GTCTTGATAG CTCTCAAGTT 1800

1801 CAAGACTTCA CTAACATGTT GCTGTCATGG TCACAGTTCT GTAAGGAGCC ACTTTCTGAG 1860

1861 CGTAATTTCA CTTTTTGGGA ATGGTTCTTT GCTGTTATGA AGGTAACCAG AGAACACCTT 1920

1921 CGTCAACCTT GGAATGATGG TTCTATCATG GGCTTTGTTG GACGTCGCCC GGCAGAGGAG 1980

1981 ATGCTGAAAA ATTCGAAAAG TGGAACGTTC CTCTTAAGAT TTTCCGACTC AGAATTGGGA 2040

2041 GGGGTTACCA TTGCATGGAT GTATGAAGAT ACTACAAAAG GTGACCAGCG GGATGTCTTC 2100

2101 ATGTTGCAGC CTTTCACAAG CAAGGCTTTT GCAATCCGTC CACTAGCTGA TGTTATTGCT 2160

2161 GACTTGAAGT ATTTGCTCTA TTTATATCCA AATGTGCCTA AAGAGCAGGC ATTTGGAAAG 2220

2221 TACTACACTC CAATGGGAGG AGAGCAGCCC ACAAATAATG GATATGTGAA ACCACATCTT 2280

2281 ATCACTCATG TACCTGGATG GTCAGTGGCT GGAGGGTCGA TGGATTCTTA TCCCAACACA 2340

2341 CCGCAACCTA TGTACCCAAT GCATGACAGC AATATGGGTG ATCCTCCGTC CGTCAGTTCC 2400

2401 AATCCCTCCG ACAGTGTCTC AACAATGCCC CCATACAACG ATACCGATTA TCCTGACATC 2460

2461 TTGGAAAATC TACCAGATAC TGACTTCACT GATATCAACC TTGACTTTCT TCAAACCAAC 2520

2521 TTCATGAAGC CCCAGTAAAA TCAGATGAAG ATTTTTTTTA ATTGTCATAC TGTATATAAG 2580

2581 CATTGTTTAT TGGATAGCCT TGCTTTTAAT ATCTTAGGAT GGTCTTTAAT TGAATTTTGC 2640

2641 ATTTAAAAAG GCAAAGAAAA AGAAGAGTGT ATTTTCAGAG GATGATGTGT AACCATAGTG 2700

2701 TCTTAGGGGT ATGCTTTAAT GTTAGGTGAA TATGAAATCG TTGTTACAAG AATTCTGCAA 2760

2761 ACGTGTATGT AGGAATTACC TTTCTCATAG ATCTTATATA AAAATGTTAA AAGGATTTCA 2820

2821 GAAATATAGC TTCATGTGTT TGATTCAATT TACAAATCAC TTAAATACAC AAAACTATTT 2880

2881 GCATTAACTG GCAAAATTTA ATCAAA 2907

**S7B Fig**


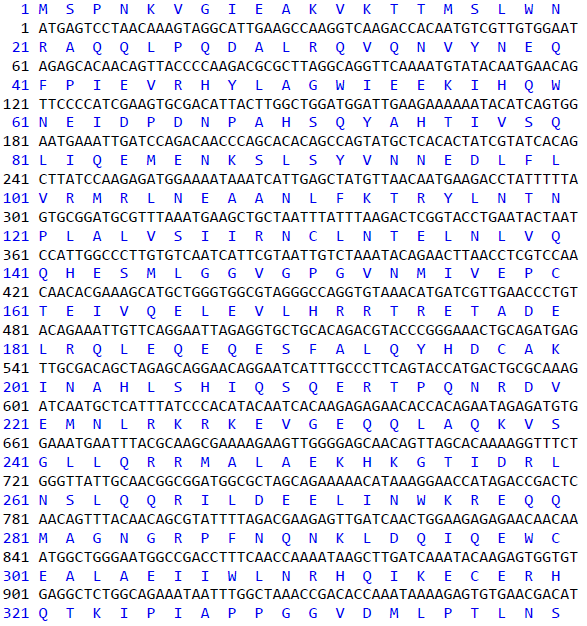


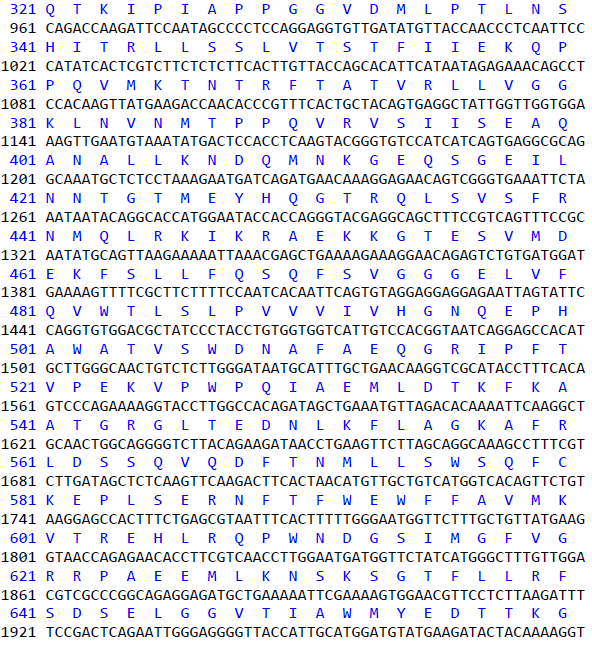


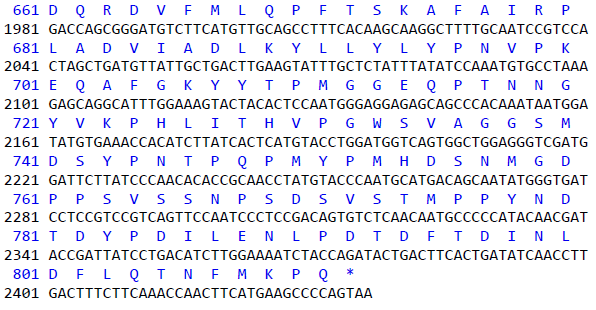


**S7 Fig**
