# Supplementary material for: Differential STAT gene expressions of Penaeus monodon and Macrobrachium rosenbergii in response to white spot syndrome virus (WSSV) and bacterial infections: Additional insight into genetic variations and transcriptomic highlights
Source: PLoS One. 2021 Oct 15;16(10):e0258655. doi: 10.1371/journal.pone.0258655 (PMC8519450; doi:10.1371/journal.pone.0258655)
Supplement: S8 Fig — A) Nucleotide sequence of Penaeus monodon STAT (PmST) obtained (full coding sequence). A total of 2491 base pair (bp) were observed for PmST, with an ORF of 2340 bp (ORF labelled in blue). B) PmST amino acid sequence numbered from N-terminus aligned with respective ORF nucleotide sequence. The protein consists of 779 amino acids (amino acid labelled in blue). (DOCX) [file pone.0258655.s008.docx]

**S8A Fig**

1 CGAACCTGTG GACTTTGTTG CAATCCTGTG GCGTCATGGC ATGGCGAGTG CGTTGATTGA 60

61 CTCTCACTTA CGCTGATTGC TGGCCAAGTG CGTATAGGAG CCCTTCACGT CGGTAAAATA 120

121 TGAGTGAAAG CACAATGTCG TTGTGGAACA GAGCACAGCA GCTTCCCGCA GATGACCTCC 180

181 GTCGGGTCCA GGGTATATAT GGTGAACAGT TCCCGATTGA GGTGCGACAC TACCTGGCAG 240

241 GATGGATAGA AGACAAGATG CAGCAATGGA ATGAAATTGA TCCAGATAAT CCAAGCCATA 300

301 GTCAGTATGC CCAGTCCCTA GTGTCCCAGC TCATACAAGA AATTGAAAAC AAAGCTCTAA 360

361 GTTATGCAAA CAATGAAGAC CTATTTCTTG TCCGTATGCG TTTAGATGAA GCTGCAACCT 420

421 CCTTCAGGAC ACGGTACCTG AACAGCAATC CCCTTGGGCT GGTGGGCATC ATACGCCAGT 480

481 GCTTGAACAC TGAACACAAT CTGGTTCAAC AGAATGAAAA TATGTTAGGA GGAGGTGTTT 540

541 CACATGCAAC AAACATGGTC ATAGAACCAT GTGCAGAAAT TGAGCAGGAG TTAAGGATAC 600

601 TTCATGAACG TACACGGGAA ACGGCAAATG AACTGAGACA CTTGGAACAA GAGCAAGAAT 660

661 CATTTGCCTT ACAATACCAT GACTGTGCAA AAATTAATGC TCACCTTTCC CACATTCAAA 720

721 GCCAGGAGAG AACGCAACAG AATCGAGAGA TGGAACAGAG CCTTCGGAGA CGGAAAGAGT 780

781 TAGGGGAGCA ACAGTTGGCG CAGAAGGTGT CAGGGCTATT GCAGCTGCGC ATGGCTCTGG 840

841 CAGATAAGCA CAAAGGTACC ATTGATCGCT TGAATAGTCT TCAGCAGCGC ATCCTAGATG 900

901 AAGAGTTAAT CAATTGGAAG AGAGACCAGC AGATGCATGG GAATGGCAAA CCTTTTAATC 960

961 CAAACAAATT AGACCAGATA CAAGAATGGT GTGAAGCACT AGCAGAAATT ATATGGCTCA 1020

1021 ATCGACACCA GATAAAAGAA TGTGAACGAC ACCAGACTAA GATACCCATA ACTCCTCCTG 1080

1081 GCGGGGTTGA CATGCTGCCA ACTCTAAATT CCCACATTAC TCGTCTTTTG TCATCACTTG 1140

1141 TTACCAGTAC TTTTATTATT GAGAAACAGC CACCCCAAGT GATGAAAACA AACACACGCT 1200

1201 TCACAGCAAC TGTCCGTTTA CTTGTGGGAG GCAAATTAAA TGTAAACATG ACACCTCCCC 1260

1261 AAGTGCGAGT CTCAATCATT AGTGAAGCCC AGGCTAATGC TCTGTTGAAG AACGACCAAA 1320

1321 TGAACAAGGG GGAACAGTCT GGTGAGATCC TGAACAACAC TGGAACTATG GAGTATAATC 1380

1381 AGACTTCGAG GCAGCTTTCA GTTAGTTTCC GTAATATGCA GCTGAGGAAG ATCAAGCGAG 1440

1441 CTGAGAAAAA GGGCACAGAG TCGGTAATGG ATGAGAAATT CTCCCTACTT TTTCAGTCAC 1500

1501 AGTTCAGTGT GGGAGGTGGA GAACTTGTTT TCCAAGTATG GACTCTGTCT TTGCCAGTTG 1560

1561 TGGTTATTGT CCATGGCAAT CAGGAGCCTC ATGCTTGGGC AACAGTGTCA TGGGATAATG 1620

1621 CATTTGCAGA ACAAGGCCGC ATTCCATTTA CTGTTCCAGA AAAGGTACCA TGGCCACAAA 1680

1681 TTGCAGACAT GTTAGACACC AAATTCAAAG CTGCCACTGG AAGGGGACTA ACAGAAGACA 1740

1741 ACCTGAAATT CCTGGCAGGA AAAGCCTTTA GGAATCCACA AGTCCAAGAT TTTACAAACA 1800

1801 TGATGCTCTC ATGGTCCCAG TTCTGCAAGG AGCCCCTGTC TGAGCGAAAC TTCACATTTT 1860

1861 GGGAGTGGTT CTTTGCAGTG ATGAAGGTTA CAAGAGAACA CCTGCGCCAG CAGTGGAACG 1920

1921 ATGGCTCCAT CATGGGTTTT GTGGGTCGAC GCCAGGCAGA AGAAATGCTT AAGAATTCCA 1980

1981 AGTCAGGCAC TTTCTTGCTC CGTGTCTCTG ACTCTGAATT AGGAGGGGTG ACAATTGCCT 2040

2041 GGATGTATGA AGATACGACT AAAGCCTGTC AACGTGATGT TTTCATGCTG CAGCCATTCA 2100

2101 CCAGTAAAGC CTTCGCCATC CGTCCTCTAG CTGATGTGAT CGCTGACTTG AATTACCTCC 2160

2161 TTTACCTATA TCCGAATGTG CCTAAGGATC AAGCCTTTGG AAAGTACTAC ACGCCCCTTG 2220

2221 GAGAGCAACA GCCCACAACT AATAATGGTT ATGTAAAGCC TCAACTAAAG ACCCATGTAC 2280

2281 CAGGATGGTC AGGTGATCCC ATGGATTCAT ATCCCAACAC ACCACAAACT ATGTATGGTG 2340

2341 GCATGGGTGG CCCTCCGTCC GTTAGTTCAA ATCCATCTGA CTGTGTCTCA ACTGACCAAA 2400

2401 AACCTACGTT GGATTCTCCT TTATTTGATG CAGCAAATGT CCTCTCAGAC TTTTCATAAG 2460

2461 TTTGCACAAC AAGCTGTATC ATTTTTGAGC T 2491

**S8B Fig**


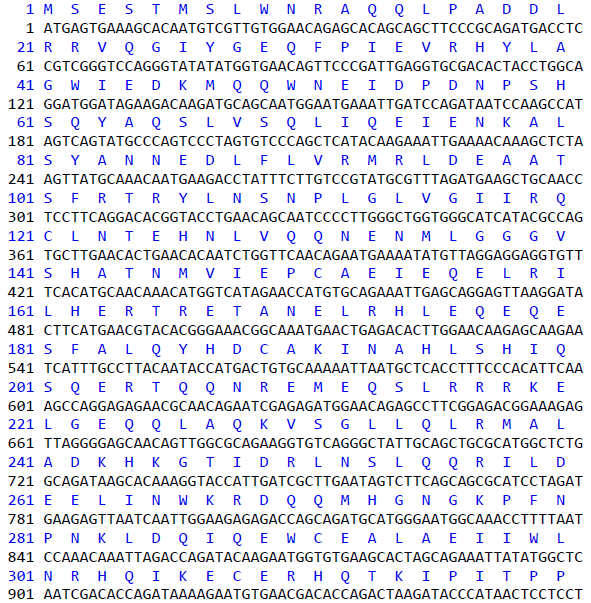


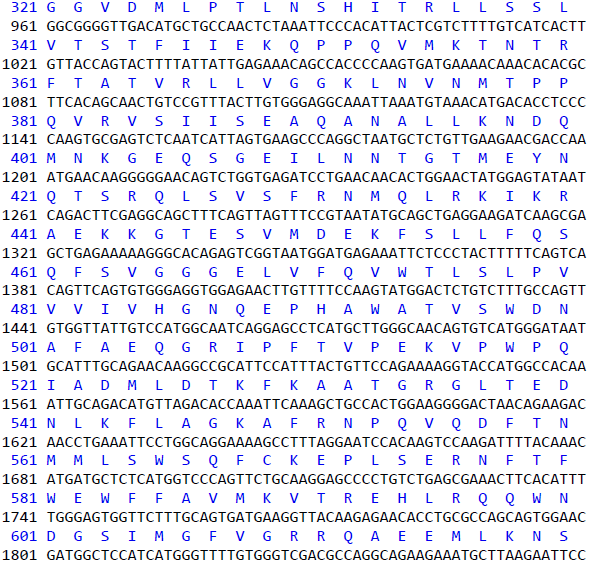

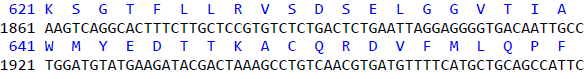


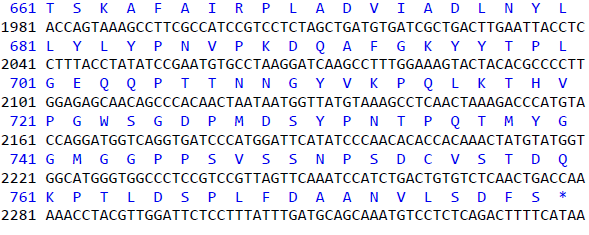


**S8 Fig**
