# Supplementary material for: Differential STAT gene expressions of Penaeus monodon and Macrobrachium rosenbergii in response to white spot syndrome virus (WSSV) and bacterial infections: Additional insight into genetic variations and transcriptomic highlights
Source: PLoS One. 2021 Oct 15;16(10):e0258655. doi: 10.1371/journal.pone.0258655 (PMC8519450; doi:10.1371/journal.pone.0258655)
Supplement: S1 Table — (DOCX) [file pone.0258655.s013.docx]

**S1 Table**

| **Gene** | **Accession Number** | **Primer/Probe Sequence** |
| --- | --- | --- |
| *MrST* | KT380661.1 | **MrST F:** 5’- ACC AAC CCT CAA TTC CCA TA -3’  **MrST R:** 5’- TTC AAC TTT CCA CCA ACC AA -3’  **MrST Probe:** 5’- CCC GTT TCA CTG CCA TAG TGA GGC -3’ |
| *M. rosenbergii* Elongation Factor 1-Alpha | EL609261.1 | **EF1-A F:** 5’- ACT GCG CTG TGT TGA TTG TAG CT -3’  **EF1-A R:** 5’- ACA ACA GTA CGT GTT CAC GGG TC -3’  **EF1-A Probe:** 5’- TAC TGG TGA GTT TGA AGC T -3’ |
| *PmST* | N/A | **LvqST2F:** 5’- GGT GAC AAT TGC CTG GAT GTA -3’  **LvqST2R:** 5’- ATC AGC TAG AGG ACG GAT GGC -3’ |
| *P. monodon* Elongation Factor 1-Alpha | MG775229.1 | **qELF F**: 5’- TAT GGT TGT CAA CTT TGC CCC -3’  **qELF R**: 5’- AAC CTC GCT TCA GAT CCT TTA C -3’ |

* N/A = Not Available
