# Supplementary material for: Differential STAT gene expressions of Penaeus monodon and Macrobrachium rosenbergii in response to white spot syndrome virus (WSSV) and bacterial infections: Additional insight into genetic variations and transcriptomic highlights
Source: PLoS One. 2021 Oct 15;16(10):e0258655. doi: 10.1371/journal.pone.0258655 (PMC8519450; doi:10.1371/journal.pone.0258655)
Supplement: S2 Table — (DOCX) [file pone.0258655.s014.docx]

**S2 Table**

| **Gene** | **Accession Number** | **Primer Sequence** |
| --- | --- | --- |
| *MrST* | **KT380661.1** | **FUMrST 1F:** 5'- TAA CAC TTC AGC CAT TTT CAC G -3'  **MrqST1R:** 5’- CTG ATG TCG TTC ACA CTC TTT -3’ |
| *MrST* | **KT380661.1** | **MrqST1F:** 5’- CAA CAA ATG GCT GGG AAT GG -3’  **MrST1R:** 5’- GAA AGT GGC TCC TTA CAG AAC -3’ |
| *MrST* | **KT380661.1** | **MrST1F:** 5’- GTC ATT GTC CAC GGT AAT C -3’  **FUMrST 1R:** 5'- TTT GAT TAA ATT TTG CCA GTT AAT GC -3' |
| *PmST* | N/A | **FUPmST 1F:** 5'- CGA CCC TGT GAA CTT TGT TGC -3'  **PMS 1R:** 5’- CAC ACC ATT CTT GTA TCT GGT CT -3’ |
| *PmST* | N/A | **PMS 2F:** 5’- TGG AAG AGA GAC CAG CAG AT -3’  **PMS 2R:** 5’- CCA TGA CAC TGT TGC CCA AG -3’ |
| *PmST* | N/A | **PMS 3F:** 5’- TTA TTG AGA AAC AGC CAC CCC AA -3’  **FUPmST 1R:** 5'- CCA TAC TGT GGT TTA TTT GAA GC -3' |

*N/A= Not Available
