# Supplementary material for: Differential STAT gene expressions of Penaeus monodon and Macrobrachium rosenbergii in response to white spot syndrome virus (WSSV) and bacterial infections: Additional insight into genetic variations and transcriptomic highlights
Source: PLoS One. 2021 Oct 15;16(10):e0258655. doi: 10.1371/journal.pone.0258655 (PMC8519450; doi:10.1371/journal.pone.0258655)
Supplement: S4 Table — (DOCX) [file pone.0258655.s016.docx]

**S4 Table**

**(A)**

| **ANOVA** | | | | | |
| --- | --- | --- | --- | --- | --- |
| **Relative Gene Expression** | | | | | |
|  | **Sum of Squares** | **df** | **Mean Square** | **F** | **Sig.** |
| Between Groups | 121.007 | 5 | 24.201 | 45.138 | <0.001 |
| Within Groups | 6.434 | 12 | 0.536 |  |  |
| Total | 127.441 | 17 |  |  |  |

**(B)**

| **Relative Gene Expression** | | | | | |
| --- | --- | --- | --- | --- | --- |
| **Duncan^a^** | | | | | |
| **Time Post-Infection (Hours)** | **N** | **Subset for alpha = 0.05** | | | |
|  |  | **a** | **b** | **c** | **d** |
| 0 | 3 | 1.23788 |  |  |  |
| 3 | 3 | 2.27247 |  |  |  |
| 6 | 3 |  | -1.76647 |  |  |
| 48 | 3 |  | -1.12550 |  |  |
| 12 | 3 |  |  | -3.26233 |  |
| 24 | 3 |  |  |  | -5.42868 |
| Sig. |  | 0.109 | 0.305 | 1.000 | 1.000 |
| Means for groups in homogeneous subsets are displayed. | | | | | |
| a. Uses Harmonic Mean Sample Size = 3.000. | | | | | |
