# Supplementary material for: Differential STAT gene expressions of Penaeus monodon and Macrobrachium rosenbergii in response to white spot syndrome virus (WSSV) and bacterial infections: Additional insight into genetic variations and transcriptomic highlights
Source: PLoS One. 2021 Oct 15;16(10):e0258655. doi: 10.1371/journal.pone.0258655 (PMC8519450; doi:10.1371/journal.pone.0258655)
Supplement: S6 Table — (DOCX) [file pone.0258655.s018.docx]

**S6 Table**

**A)**

| **ANOVA** | | | | | |
| --- | --- | --- | --- | --- | --- |
| **Relative Gene Expression** | | | | | |
|  | **Sum of Squares** | **df** | **Mean Square** | **F** | **Sig.** |
| Between Groups | 8.501 | 5 | 1.700 | 8.375 | 0.001 |
| Within Groups | 2.436 | 12 | 0.203 |  |  |
| Total | 10.937 | 17 |  |  |  |

**B)**

| **Relative Gene Expression** | | | | |
| --- | --- | --- | --- | --- |
| **Duncan^a^** | | | | |
| **Time Post-Infection (Hours)** | **N** | **Subset for alpha = 0.05** | | |
|  |  | **a** | **b** | **c** |
| 48 | 3 | 1.27258 |  |  |
| 0 | 3 | 1.62174 | 1.62174 |  |
| 12 | 3 |  | 2.29755 | 2.29755 |
| 6 | 3 |  |  | 2.49970 |
| 3 | 3 |  |  | 3.05898 |
| 24 | 3 |  |  | 3.13869 |
| Sig. |  | 0.361 | 0.091 | 0.055 |
| Means for groups in homogeneous subsets are displayed. | | | | |
| a. Uses Harmonic Mean Sample Size = 3.000. | | | | |
