# Supplementary material for: Differential STAT gene expressions of Penaeus monodon and Macrobrachium rosenbergii in response to white spot syndrome virus (WSSV) and bacterial infections: Additional insight into genetic variations and transcriptomic highlights
Source: PLoS One. 2021 Oct 15;16(10):e0258655. doi: 10.1371/journal.pone.0258655 (PMC8519450; doi:10.1371/journal.pone.0258655)
Supplement: S7 Table — (DOCX) [file pone.0258655.s019.docx]

**S7 Table**

| **Annotated Identity** | **Gene ID** | **Differential Expression with Log2 Fold Change** | **Source** |
| --- | --- | --- | --- |
| **Stress DEGs** | | | |
| Inositol 1,4,5-trisphosphate receptor | WM: Unigene16943 | (Up, 3.117) | SP (P29993) |
|  | VM: Unigene16943 | (Up, 3.151) | SP (P29993) |
|  | WP: Unigene23317 | (Down, -4.235) | SP (P29993) |
|  | AP: Unigene8201 | T3 (Up, 1.395),  T6 (*, 0.745),  T24 (Up, 1.203) | SP (P29993) |
| Dopamine N-acetyltransferase | WM: N/D | - | - |
|  | VM: N/D | - | - |
|  | WP: Unigene6105 | (Up, 1.476) | SP (Q94521) |
|  | AP: Unigene2599 | T3 (Up, 1.174),  T6 (*, 0.937),  T24 (*, -0.213) | KEGG (K00669) |
| Hsp90 | WM: CL4309.Contig3 | (Up, 2.027) | NR (AGC54636.1) |
|  | VM: CL4309.Contig3 | (Up, 1.936) | NR (AGC54636.1) |
|  | WP: Unigene21424 | (Up, 1.496) | NR (AB520827.1) |
|  | AP: Unigene3244 | T3 (Up, 5.157),  T6 (Up, 2.983),  T24 (Up, 4.972) | NR (ABM54577.1) |
| Apoptosis-stimulating of p53 protein 1 | WM: N/D | - | - |
|  | VM: N/D | - | - |
|  | WP: Unigene20777 | (Down, -1.688) | SP (Q8CG79) |
|  | AP: CL1336.Contig2 | T3 (Up, 5.478),  T6 (*, -1.663),  T24 (Up, 5.482) | KEGG (K17554) |
| Caspase | WM: CL2405.Contig1 | (Down, -1.546) | KEGG (K04489) |
|  | VM: CL2405.Contig1 | (Up, 2.0599) | KEGG (K04489) |
|  | WP: Unigene2269 | (Down, -2.895) | KEGG (K04489) |
|  | AP: CL2405.Contig1 | T3 (Down, -1.231),  T6 (*, 0.865),  T24 (*, 0.0314) | KEGG (K04489) |
| Apoptosis-inducing factor (AIF) | WM: N/D | - | - |
|  | VM: N/D | - | - |
|  | WP: N/D | - | - |
|  | AP: CL39.Contig2 | T3 (Down, -1.880),  T6 (*, -0.214),  T24 (Up, 1.883) | KEGG (K04727) |
| **Endocrine DEGs** | | | |
| Mitochondrial coenzyme A transporter | WM: CL4093.Contig2 | (Up, 10.872) | SP (Q0P483) |
|  | VM: CL4093.Contig2 | (Up, 11.017) | SP (Q0P483) |
|  | WP: Unigene2818 | (Down, -1.554) | SP (P0C546) |
|  | AP: CL247.Contig5 | T3 (Up, 8.082),  T6 (Up, 7.267),  T24 (*, 4.460) | SP (P0C546) |
| ATP binding cassette transmembrane transporter | WM: CL1533.Contig3 | (Up, 1.161) | KEGG (K05679) |
|  | VM: CL1533.Contig3 | (Down, -2.152) | KEGG (K05679) |
|  | WP: N/D | - | - |
|  | AP: Unigene31054 | T3 (*, -2.807),  T6 (*, 0.652),  T24 (Up, 4.226) | KEGG (K05679) |
| Trehalose transporter | WM: CL2313.Contig2 | (Down, -9.485) | KEGG (K14258) |
|  | VM: CL2313.Contig2 | (Down, -6.020) | KEGG (K14258) |
|  | WP: Unigene28086 | (Up, 3.072) | KEGG (K14258) |
|  | AP: Unigene16953 | T3 (Up, 8.428),  T6 (*, 1.807),  T24 (Up, 6.170) | KEGG (K14258) |
| Polysaccharide lyase | WM: Unigene13470 | (Up, 3.620) | NR (XP_004341880.1) |
|  | VM: Unigene13470 | (Up, 4.900) | NR (XP_004341880.1) |
|  | WP: CL217.Contig4 | (Down, -2.579) | NR (XP_004341880.1) |
|  | AP: CL3669.Contig3 | T3 (Up, 12.106),  T6 (Up, 5.954),  T24 (Up, 12.121) | NR (XP_004341880.1) |
| Trypsin | WM: Unigene24022 | (Up, 3.780) | KEGG (K01312) |
|  | VM: Unigene24022 | (Up, 4.301) | KEGG (K01312) |
|  | WP: Unigene16275 | (Down, -2.663) | KEGG (K01312) |
|  | AP: Unigene8545 | T3 (Up, 10.996),  T6 (Up, 6.304),  T24 (Up, 10.766) | KEGG (K01312) |
| Peroxisomal acyl-coenzyme A oxidase | WM: CL5158.Contig3 | (Up, 1.376) | KEGG (K00232) |
|  | VM: CL5158.Contig3 | (Up, 1.192) | KEGG (K00232) |
|  | WP: Unigene6812 | (Down, -3.913) | KEGG (K00232) |
|  | AP: CL3140.Contig1 | T3 (Down, -10.917),  T6 (Down, -10.917),  T24 (Down, -8.595) | KEGG (K00232) |
| **Immune DEGs** | | | |
| Transglutaminase | WM: Unigene6536 | (Up, 3.053) | KEGG (K05619) |
|  | VM: Unigene6536 | (Up, 3.481) | KEGG (K05619) |
|  | WP: Unigene12697 | (Up, 1.093) | KEGG (K05619) |
|  | AP: Unigene9818 | T3 (Up, 1.874),  T6 (*, 1.247),  T24 (Up, 1.731) | KEGG (K05619) |
| C-type Lectin | WM: CL1600.Contig1 | (Up, 1.504) | NR (ADW08727.1) |
|  | VM: Unigene9635 | (Up, 1.147) | NR (AAZ29608.1) |
|  | WP: Unigene17580 | (Down, -1.271) | NR (AAZ29608.1) |
|  | AP: Unigene5450 | T3 (Up, 1.766),  T6 (Up, 1.841),  T24 (Up, 2.097) | NR (AAZ29608.1) |
| HMGB | WM: Unigene16516 | (Down, -1.388) | NR (ADQ43367.1) |
|  | VM: N/D | - | - |
|  | WP: CL2495.Contig2 | (Up, 1.745) | NR (ADQ43367.1) |
|  | AP: Unigene17462 | T3 (Up, 2.570),  T6 (*, 0.926),  T24 (Up, 2.719) | NR (ADQ43366.1) |
| ALF1 | WM: Unigene4120 | (Up, 1.666) | NT (FJ429306.1) |
|  | VM: Unigene4120 | (Up, 4.132) | NT (FJ429306.1) |
|  | WP: CL1912.Contig1 | (Up, 1.004) | NT (EF523560.1) |
|  | AP: Unigene16331 | T3 (Up, 2.215),  T6 (Up, 3.051),  T24 (Up, 3.478) | NT (EF523560.1) |
| ALF3 | WM: Unigene22936 | (Up, 2.158) | NR (AFW04306.1) |
|  | VM: Unigene22936 | (Up, 2.621) | NR (AFW04306.1) |
|  | WP: Unigene228 | (Up, 2.306) | NR (ABP73289.1) |
|  | AP: CL1926.Contig1 | T3 (Up, 8.397)  T6 (*, 5.728)  T24 (N/D) | NR (ABP73289.1) |
| proPO | WM: Unigene12734 | (Up, 4.549) | NR (AAX48010.1) |
|  | VM: Unigene12734 | (Up, 3.160) | NR (AAX48010.1) |
|  | WP: Unigene2300 | (Down, -2.281) | NR (ACJ31817.1) |
|  | AP: Unigene3184 | T3 (Up, 2.21),  T6 (Up, 2.395),  T24 (*, 0.602) | NR (AGI42860.1) |
| Superoxide Dismutase | WM: Unigene19405 | (Down, -3.183) | KEGG (K04565) |
|  | VM: Unigene19405 | (Up, 1.6117) | KEGG (K04565 |
|  | WP: CL1200.Contig2 | (Down, -2.365) | KEGG (K04565) |
|  | AP: Unigene3737 | T3 (Up, 3.826),  T6 (*, -0.198),  T24 (*, 0.0721) | KEGG (K04565) |
| Glutathione Peroxidase | WM: Unigene26204 | (Up, 1.858) | KEGG (K00432) |
|  | VM: N/D | - | - |
|  | WP: Unigene11417 | (Up, 2.0129) | KEGG (K00432) |
|  | AP: Unigene6270 | T3 (Up, 6.298),  T6 (*, -0.567),  T24 (*, 0.787) | KEGG (K00432) |
| Catalase | WM: CL5831.Contig2 | (Up, 3.566) | KEGG (K03781) |
|  | VM: CL2477.Contig1 | (Up, 1.609) | NT (JX162772.1) |
|  | WP: Unigene15932 | (Down, -1.005) | KEGG (K03781) |
|  | AP: Unigene19769 | T3 (Down, -1.271),  T6 (*, 0.127),  T24 (Down, -1.370689755) | NT (KR908786.1) |
| **Signalling DEGs** | | | |
| Ceramide synthase | WM: CL2309.Contig1 | (Up, 2.2689) | KEGG (K04710) |
|  | VM: CL2309.Contig1 | (Up, 3.5367) | KEGG (K04710) |
|  | WP: CL50.Contig1 | (Down, -1.244) | KEGG (K04710) |
|  | AP: CL1495.Contig1 | T3 (Up, 11.053),  T6 (Up, 10.143),  T24 (Up, 9.562) | KEGG (K04710) |
| Calcium-activated chloride channel regulator | WM: CL2902.Contig2 | (Up, 1.720) | KEGG (K05030) |
|  | VM: CL2902.Contig2 | (Up, 1.528) | KEGG (K05030) |
|  | WP: Unigene22512 | (Down, -4.235) | KEGG (K05030) |
|  | AP: Unigene14387 | T3 (Up, 9.613),  T6 (Down, -5.615),  T24 (Up, 1.652) | KEGG (K05030) |
| Inward rectifier potassium channel | WM: CL5263.Contig2 | (Up, 1.532) | KEGG (K05330) |
|  | VM: CL5263.Contig2 | (Up, 3.251) | KEGG (K05330) |
|  | WP: Unigene8936 | (Up, 1.575) | KEGG (K05330) |
|  | AP: CL2046.Contig3 | T3 (Down, -7.140),  T6 (Up, 1.609),  T24 (*, 0.554) | KEGG (K05330) |
| STAT | WM: Unigene25562 | (Up, 2.788) | KEGG (K11224) |
|  | VM: Unigene25562 | (Up, 2.846) | KEGG (K11224) |
|  | WP: CL1273.Contig1 | (Down, -1.457) | KEGG (K11224) |
|  | AP: Unigene21346 | T3 (Up, 2.569),  T6 (Up, 1.693),  T24 (Up, 2.655) | KEGG (K11224) |
| IMD | WM: N/D | - | - |
|  | VM: N/D | - | - |
|  | WP: N/D | - | - |
|  | AP: Unigene18449 | T3 (Up, 6.950),  T6 (*, 0),  T24 (Up, 5.044) | NR (ACL37048.1) |
| TBK1 | WM: CL523.Contig3 | (Up, 1.997) | KEGG (K05410) |
|  | VM: CL523.Contig3 | (Up, 1.787) | KEGG (K05410) |
|  | WP: Unigene11183 | (Down, -1.040) | KEGG (K05410) |
|  | AP: Unigene7561 | T3 (*, -0.0590),  T6 (*, -0.950),  T24 (*, -0.690) | KEGG (K05410) |
| **Structural DEGs** | | | |
| Actin | WM: CL6026.Contig2 | (Up, 3.463) | NT (AF100986.1) |
|  | VM: CL6026.Contig2 | (Up, 4.931) | NT (AF100986.1) |
|  | WP: CL171.Contig12 | (Up, 1.023) | NT (AF100986.1) |
|  | AP: CL881.Contig12 | T3 (Up, 1.789),  T6 (Down, -1.399),  T24 (*, 0.612) | NT (AF100986.1) |
| Ankyrin | WM: CL2980.Contig2 | (Up, 4.006) | KEGG (K10380) |
|  | VM: CL2980.Contig2 | (Up, 3.717) | KEGG (K10380) |
|  | WP: CL137.Contig15 | (Up, 1.388) | KEGG (K10380) |
|  | AP: CL1999.Contig2 | T3 (Up, 10.203),  T6 (Up, 11.989),  T24 (Up, 11.002) | KEGG (K10380) |

[WM: WSSV-infected *M. rosenbergii*; VM: *V. parahaemolyticus*-infected *M. rosenbergii*; WP: WSSV-infected *P. monodon* at 12 dpi; AP: *Vp*_AHPND_-infected *P. monodon*; T3: 3 hpi; T6: 6 hpi; T24: 24 hpi]

N/D or *: Non-Differential
